# Pharmacogenetics and Precision Medicine Approaches for the Improvement of COVID-19 Therapies

**DOI:** 10.3389/fphar.2022.835136

**Published:** 2022-02-18

**Authors:** Mohitosh Biswas, Nares Sawajan, Thanyada Rungrotmongkol, Kamonpan Sanachai, Maliheh Ershadian, Chonlaphat Sukasem

**Affiliations:** ^1^ Division of Pharmacogenomics and Personalized Medicine, Department of Pathology, Faculty of Medicine Ramathibodi Hospital, Mahidol University, Bangkok, Thailand; ^2^ Laboratory for Pharmacogenomics, Somdech Phra Debaratana Medical Center (SDMC), Ramathibodi Hospital, Bangkok, Thailand; ^3^ Department of Pharmacy, University of Rajshahi, Rajshahi, Bangladesh; ^4^ Department of Pathology, School of Medicine, Mae Fah Luang University, Chiang Rai, Thailand; ^5^ Structural and Computational Biology Research Unit, Department of Biochemistry, Faculty of Science, Chulalongkorn University, Bangkok, Thailand; ^6^ Program in Bioinformatics and Computational Biology, Graduate School, Chulalongkorn University, Bangkok, Thailand; ^7^ Pharmacogenomics and Precision Medicine, The Preventive Genomics and Family Check-up Services Center, Bumrungrad International Hospital, Bangkok, Thailand; ^8^ MRC Centre for Drug Safety Science, Department of Pharmacology and Therapeutics, Institute of Systems, Molecular and Integrative Biology, University of Liverpool, Liverpool, United Kingdom

**Keywords:** COVID-19, pathogenesis and severity, repurposed drugs, pharmacogenetics, molecular docking, drug-drug interactions, drug-herb interactions, precision medicine

## Abstract

Many drugs are being administered to tackle coronavirus disease 2019 (COVID-19) pandemic situations without establishing clinical effectiveness or tailoring safety. A repurposing strategy might be more effective and successful if pharmacogenetic interventions are being considered in future clinical studies/trials. Although it is very unlikely that there are almost no pharmacogenetic data for COVID-19 drugs, however, from inferring the pharmacokinetic (PK)/pharmacodynamic(PD) properties and some pharmacogenetic evidence in other diseases/clinical conditions, it is highly likely that pharmacogenetic associations are also feasible in at least some COVID-19 drugs. We strongly mandate to undertake a pharmacogenetic assessment for at least these drug–gene pairs (atazanavir–*UGT1A1*, *ABCB1*, *SLCO1B1*, *APOA5*; efavirenz–*CYP2B6*; nevirapine–*HLA*, *CYP2B6*, *ABCB1*; lopinavir–*SLCO1B3*, *ABCC2*; ribavirin–*SLC28A2*; tocilizumab–*FCGR3A*; ivermectin–*ABCB1*; oseltamivir–*CES1*, *ABCB1*; clopidogrel–*CYP2C19*, *ABCB1*, warfarin–*CYP2C9*, *VKORC1*; non-steroidal anti-inflammatory drugs (NSAIDs)–*CYP2C9*) in COVID-19 patients for advancing precision medicine. Molecular docking and computational studies are promising to achieve new therapeutics against SARS-CoV-2 infection. The current situation in the discovery of anti-SARS-CoV-2 agents at four important targets from *in silico* studies has been described and summarized in this review. Although natural occurring compounds from different herbs against SARS-CoV-2 infection are favorable, however, accurate experimental investigation of these compounds is warranted to provide insightful information. Moreover, clinical considerations of drug–drug interactions (DDIs) and drug–herb interactions (DHIs) of the existing repurposed drugs along with pharmacogenetic (e.g., efavirenz and *CYP2B6*) and herbogenetic (e.g., andrographolide and *CYP2C9*) interventions, collectively called multifactorial drug–gene interactions (DGIs), may further accelerate the development of precision COVID-19 therapies in the real-world clinical settings.

## 1 Introduction for Pharmacogenetics of COVID-19 Treatment

At the end of 2019, a novel coronavirus, severe acute respiratory syndrome coronavirus 2 (SARS-CoV-2) started as an emerging pathogen for humans, first appeared in Wuhan, China, in December 2019. This novel virus causes coronavirus disease 2019 (COVID-19), named by the WHO on 11 February 2020, and it has been characterized as a pandemic on 11 March 2020. COVID-19 has become the leading cause of death globally, resulting in huge economic and social disruption internationally (Hodgson et al., 2021). As of 10 September 2021 as declared by the WHO, over 223 million confirmed cases of SARS-CoV-2 infection have been detected globally in which ∼4.6 million deaths occurred (WHO, 2021). It is alarming that still now considerably a large number of patients are dying due to COVID-19. The existence of this pandemic virus had been confirmed in over 200 countries or territories, indicating that corona virus was exponentially spread out throughout the world.

One of the leading causes of morbidity and mortality might be the adverse drug reactions (ADRs) associated with current medications administered for the management of COVID-19 since the mortality rate was significantly higher in COVID-19 patients with multiple comorbidities and particularly in older patients (Manjaly Thomas et al., 2020; Ramírez et al., 2020; Biswas et al., 2021a; Falcão et al., 2021; Melo et al., 2021; Rezaee et al., 2021). Polypharmacy is highly predictable in multiple comorbid patients, and also, age-related degradation of organ function in older patients is placing them highly vulnerable to drug–drug interactions (DDIs) and consequently the most notorious ADRs or toxicities of the COVID-19 therapeutics. A recent pharmacovigilance study conducted in Spain reported the 4.75-fold higher incidence of severe ADRs in the COVID-19 patients compared to non-COVID-19 patients, in which the prevalence of severe ADRs was the highest with tocilizumab (59.8%) followed by dexketoprofen (13.9%), azithromycin (8.4%), dexamethasone (7.6%), lopinavir–ritonavir (7.4%), and chloroquine (CQ)/hydroxychloroquine (HCQ) (6.9%) (Ramírez et al., 2020). Another pharmacovigilance study conducted in Brazil with 402 COVID-19 patients indicated that chloroquine (CQ) (OR = 5.4; 95% CI: 1.9–15.6) and HCQ (OR = 2.1; 95% CI: 1.2–3.6) were the only culprit drugs associated with severe ADRs (Melo et al., 2021). A prospective observational study identified a total of 102 ADRs in 149 COVID-19 patients where the incidence of ADRs was significantly higher in patients who have taken HCQ than in the patients who have taken remdesivir (RDV) (47.5 vs. 12.5%; *p* < 0.001), as evidenced recently (Falcão et al., 2021). This is consistent with a predictive study showing that at least 329 DDIs are feasible in patients taking HCQ, and at the very least, 29 severe DDIs were identified from different reputed international interaction resources, predicted to cause severe toxicity of HCQ (Biswas and Roy, 2021). A hospital-based pharmacovigilance study conducted in China identified ∼38% ADRs in COVID-19 patients, where drug-induced gastrointestinal disorders were 23% and liver system disorders were ∼14%. These ADRs were mainly associated with the use of lopinavir/ritonavir (∼64%) and umifenovir (∼18%). Multivariate logistic analysis indicated that the number of drugs used while COVID-19 patients were staying in the hospital was one of the strongest independent risk factors for these ADRs (OR: 3.17; 95% CI 1.60–6.27; *p* = 0.001), as reported in this observational study (Sun et al., 2020).

Although there are no specific therapeutic recommendations for treating COVID-19, however, many off-label drugs are being currently administered for the management of COVID-19 and severe ADRs; for example, QT prolongation, cardiac arrhythmias, thrombosis, retinopathy, hepatotoxicity, and increased risk of infection due to DDIs are feasible in these patients as evidenced and suggested elsewhere (Biswas et al., 2020a; Lemaitre et al., 2020; Manjaly Thomas et al., 2020; Ramírez et al., 2020; Sun et al., 2020; Biswas and Roy, 2021; Falcão et al., 2021; Melo et al., 2021; Rezaee et al., 2021).

While it is increasingly true of DDIs for COVID-19 therapeutics, it is likely that the cytochrome P450 (CYP) enzymes or transporter proteins affecting the pharmacokinetics (PK) or pharmacodynamic (PD) properties were mostly involved in the reported and predicted DDIs of drugs used in the treatment of COVID-19. However, the genetic variants modulating the PK/PD profiles of COVID-19 drugs regulating the safety or effectiveness are not clinically studied yet, posing serious scarcity of pharmacogenomic data in the literature.

The PK and PD properties of the COVID-19 drugs are very potential factors to explore the pharmacogenetics association study; however, it appears that drug-developing authorities and scientists did not consider the pharmacogenetics interference in drug response variability, which could affect either the safety/effectiveness of COVID-19 drugs or the severity of COVID-19 progression. In this review, we will discuss in detail the pharmacogenetics of COVID-19 therapeutics with a particular focus on drugs targeting SARS-CoV-2 life cycle, drug–drug interactions (DDIs), and drug–herb interactions (DHIs) potentially affecting the pharmacogenetic interventions. We will also discuss some of the genetic variants potentially affecting the severity of COVID-19 progression.

## 2 Virology of SARS-CoV-2 and its Entry Into Human Cells

**FIGURE 1:**
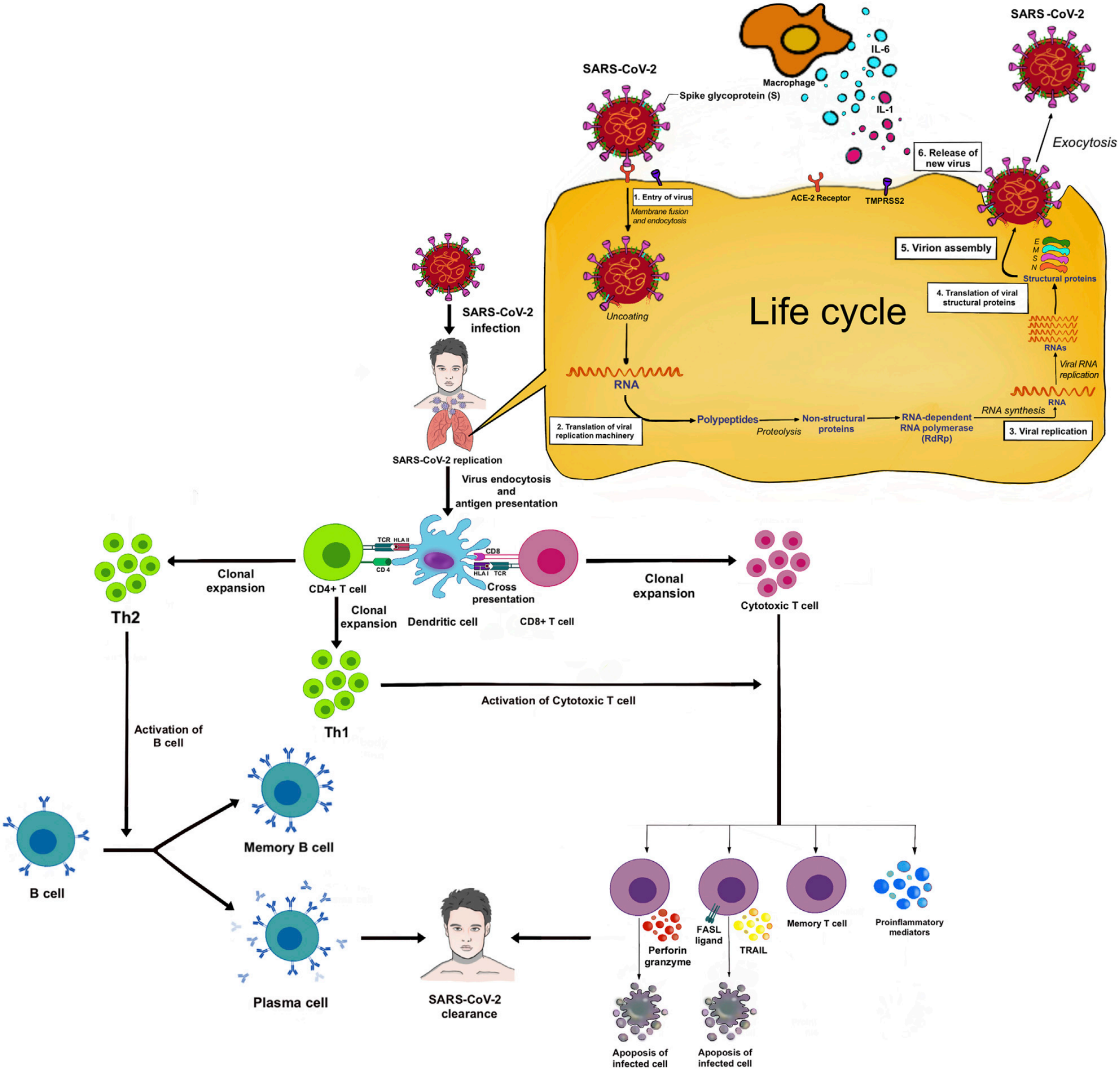
SARS-CoV-2 entry and human immune response.

SARS-CoV-2 is a positive-sense, single-stranded RNA-enveloped virus in the *Betacoronavirus* genus (Attaway et al., 2021). Bats and pangolins may be the animal hosts of SARS-CoV-2 as there is a >90% gene homology to SARS-CoV-2 found to infect humans (Hu et al., 2021a). Currently, it remains unclear if SARS-CoV-2 was directly transferred from bat/pangolins to humans or an intermediate host was required for transmission. In light of the current pandemic, researchers first compared SARS-CoV-2 with the previous endemic SARS-CoV (2002–2003) and MERS-CoV (2012) (da Costa et al., 2020). SARS-CoV-2 propagates and migrates down the respiratory tract along the conducting airways. The entry process of SARS-CoV-2 into host cells is *via* the binding of the S protein to the ACE2 receptor (Figure 1).

The virion releases its RNA. Some RNA is translated into proteins by the host cell’s machinery. Proteins and RNA are assembled into a new virion in the Golgi and released. ACE2 receptors are highly expressed in the upper respiratory tract of humans (Lan et al., 2020). Proteolytic cleavage of the S protein by serine proteases including transmembrane protease serine 2 (TMPRSS2), cathepsin L, and furin is required for binding to the ACE2 receptor (Wang et al., 2020b). In the lower respiratory tract, type II pneumocytes and alveolar macrophages also express ACE2 receptors and can be infected, and release several chemokines/cytokines. Once the virus attaches to the host cell receptors, it undergoes endocytosis, viral maturation, replication, and release of more virus within the cytoplasm of the host cell. SARS-CoV-2 infection begins with viral replication and partially avoids host recognition during the initial infection and before the host innate response is enabled (Bergmann and Silverman, 2020).

Angiotensin-converting enzyme 2 (ACE2) functions as a master regulator of the renin-angiotensin system (RAS) mainly by converting Ang (angiotensin) I and Ang II into Ang 1–9 and Ang 1–7, respectively. The ACE2 system is a critical protective pathway against heart failure, myocardial infarction, and hypertension, and against lung disease and diabetes mellitus. ACE2 is widely expressed, including in the lungs, cardiovascular system, gut, kidneys, central nervous system, and adipose tissue. ACE2 has recently been identified as the SARS-CoV-2 receptor (Kuhn et al., 2004). The loss of ACE2 function following binding by SARS-CoV-2 is driven by endocytosis and activation of proteolytic cleavage. Ang II levels elevate with increased activity of angiotensin 1 receptors (AT1R) at the cost of ACE2/Ang 1–7-driven pathways, leading to adverse fibrosis, hypertrophy, increased reactive oxygen species (ROS), vasoconstriction, and gut dysbiosis. ADAM17 (a disintegrin and metalloproteinase 17)-mediated proteolytic cleavage of ACE2 is upregulated by endocytosed SARS-CoV-2 spike proteins. The activation of the AT1R by elevated Ang II levels also further increases ADAM17 activity. ADAM17 correspondingly also cleaves its primary substrate releasing soluble TNF-α (tumor necrosis factor-α) into the extracellular region where it has auto- and paracrine functionality. TNF-α activation of its tumor necrosis factor receptor (TNFR) represents a third pathway elevating ADAM17 activity (Gheblawi et al., 2020). TNF-α along with systemic cytokines released due to SARS-CoV-2 infection can lead to a cytokine storm.

## 3 Immune Response, Pathogenesis, and Clinical Manifestation of COVID-19

**FIGURE 2:**
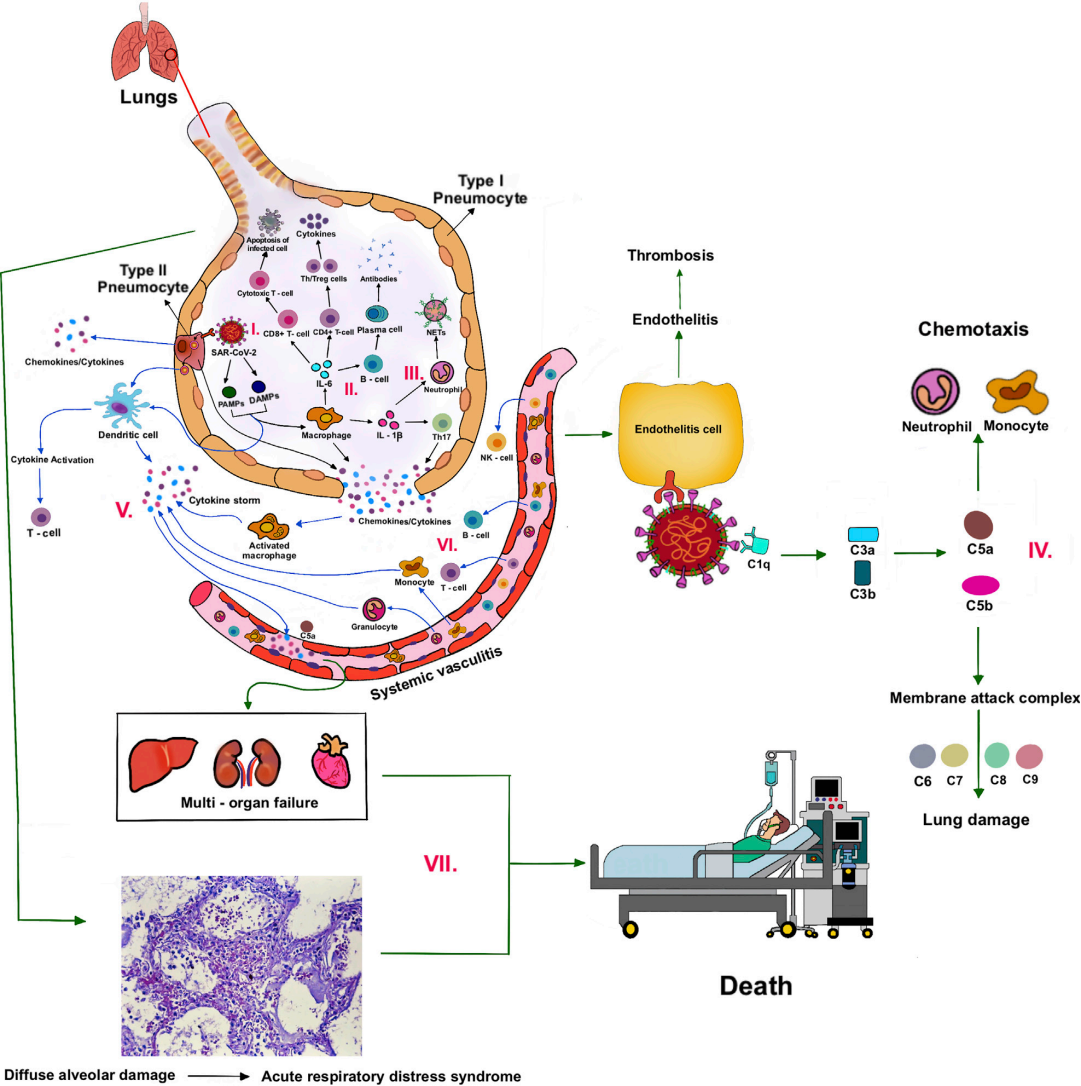
Cytokine storm and pathogenesis of COVID-19.

The cells of the airway epithelium are the first line of defense (innate immune system), providing a mechanical barrier (mucociliary escalator) that expels particles and pathogens *via* cilia, mucus, and induced coughing. This barrier includes cells of the pulmonary epithelium, alveolar macrophages (AMs), and dendritic cells (DCs). The AMs and DCs express pattern recognition receptors (PRRs), including Toll-like receptors (TLRs) and RIG-I-like receptors (RLRs), that can detect molecules from pathogens (pathogen-associated molecular patterns—PAMPs) or molecules released from damaged cells (damage or danger-associated molecular patterns—DAMPs) (Figure 2–I).

Upon recognition, these sensors recruit the adaptor proteins, MyD88 and MAVS, respectively, and induce downstream signaling. Ultimately, this leads to the activation of the transcription factors, IRF3/7 and NF-κB, and the subsequent production of type I interferons (IFN-α and IFN-β) and pro-inflammatory cytokines (e.g., IL-6 and TNF-α), respectively. Additionally, the virus is thought to activate the inflammasome sensor, NLRP3, resulting in the secretion of the highly inflammatory cytokine IL-1β and the induction of pyroptosis, an inflammatory form of cell death (Lim et al., 2016).

T cells and B cells are activated (adaptive immune system) by antigen presentation and cytokines from DCs and AMs, and activation of the complement system. IL-6 promotes the differentiation to cytotoxic T cells, helper T cells (Th), and plasma cells. IL-1β promotes the differentiation of Th17, which functions by stimulating neutrophil recruitment and inflammation (Figure 2–II). Cytotoxic T cells play a crucial role in SARS-CoV-2 clearance due to their ability to selectively eliminate virus-infected cells by inducing apoptosis *via* ligands such as Fas ligand (FasL) and tumor necrosis factor-related apoptosis-inducing ligand (TRAIL), and perforin/granzyme-mediated pathway (Ranasinghe et al., 2016; Huang et al., 2019). Th1 helps in the activation of cytotoxic T cells. Th2 activates B cells to produce antibodies and become plasma cells. These antibodies contribute to SARS-CoV-2 clearance. There are memory T and B cells that can help against the recurrent infection (Figure 1).

Neutrophils are attracted by chemokines/cytokines swarm to the site of infection. Subsequently, activated neutrophils undergo degranulation and (neutrophil extracellular traps) NET formation, releasing intracellular DAMPs, DNA, histones, and neutrophil elastase that activates the PRRs of surrounding immune and non-immune cells to induce cytokine secretion (Figure 2–III). Neutrophils and NETs drive necro-inflammation in COVID-19 (Barnes et al., 2020). The extracellular DNA released by NETs activates platelets, and aggregated NETs provide a scaffold for binding of erythrocytes and activated platelets that promote thrombus formation (Mozzini and Girelli, 2020).

In a later phase of SARS-CoV-2 infection, the complement system will be triggered *via* antibodies bound to the virus (Noris et al., 2020). C3 can be converted into C3a and C3b. C3b mediates pathogen opsonization and activates the conversion of C5 into C5a and C5b. C5b mediates the formation of the membrane attack complex (MAC), which leads to cell lysis. C3a and C5a promote immune cell recruitment to the site of infection (Figure 2–IV).

Excessive cytokines produced by macrophages and DCs, that is, IL-1β, IFN-I, CXCL10, CXCL11, IL-6, IP-10, and TNF-α, are called cytokine storm (Ahmed-Hassan et al., 2020) (Figure 2–V). Cytokine storm and C5a lead to the influx of immune cells (e.g., granulocytes, monocytes, T cells, B cells, and NK cells) into the infected site (Wang et al., 2015) (Figure 2–VI). The overwhelming infiltrate of immune cells causes excessive pulmonary inflammation (severe pneumonia) with destructive effects on human tissue, resulting in destabilization of endothelial cell to cell interactions, damage of vascular barrier, capillary damage, diffuse alveolar damage (DAD), pulmonary fibrosis, systemic inflammation, hyperferritinemia, hemodynamic instability, and multi-organ failure, and if left untreated, it leads to death (Ackermann et al., 2021; Chen and Pan, 2021) (Figure 2–VII). Acute respiratory distress syndrome (ARDS), as a result of DAD, leads to low oxygen saturation levels and is a major cause of mortality in COVID-19. SARS-CoV-2 also can infect the endothelial cells, causing endothelial injury, endotheliitis, and microthrombus formation in several organs, especially in alveolar capillary (Varga et al., 2020). The electron microscopy shows new vessel growth through a mechanism of intussusceptive angiogenesis, especially in patients with a long duration of hospitalization (Ackermann et al., 2020). These microangiopathies could be the factors, which are worsening the ARDS. Although the exact mechanism of ARDS in COVID-19 patients is not fully understood, the excessive production of pro-inflammatory cytokines is considered to be one of the major contributing factors (Chen et al., 2020).

**FIGURE 3:**
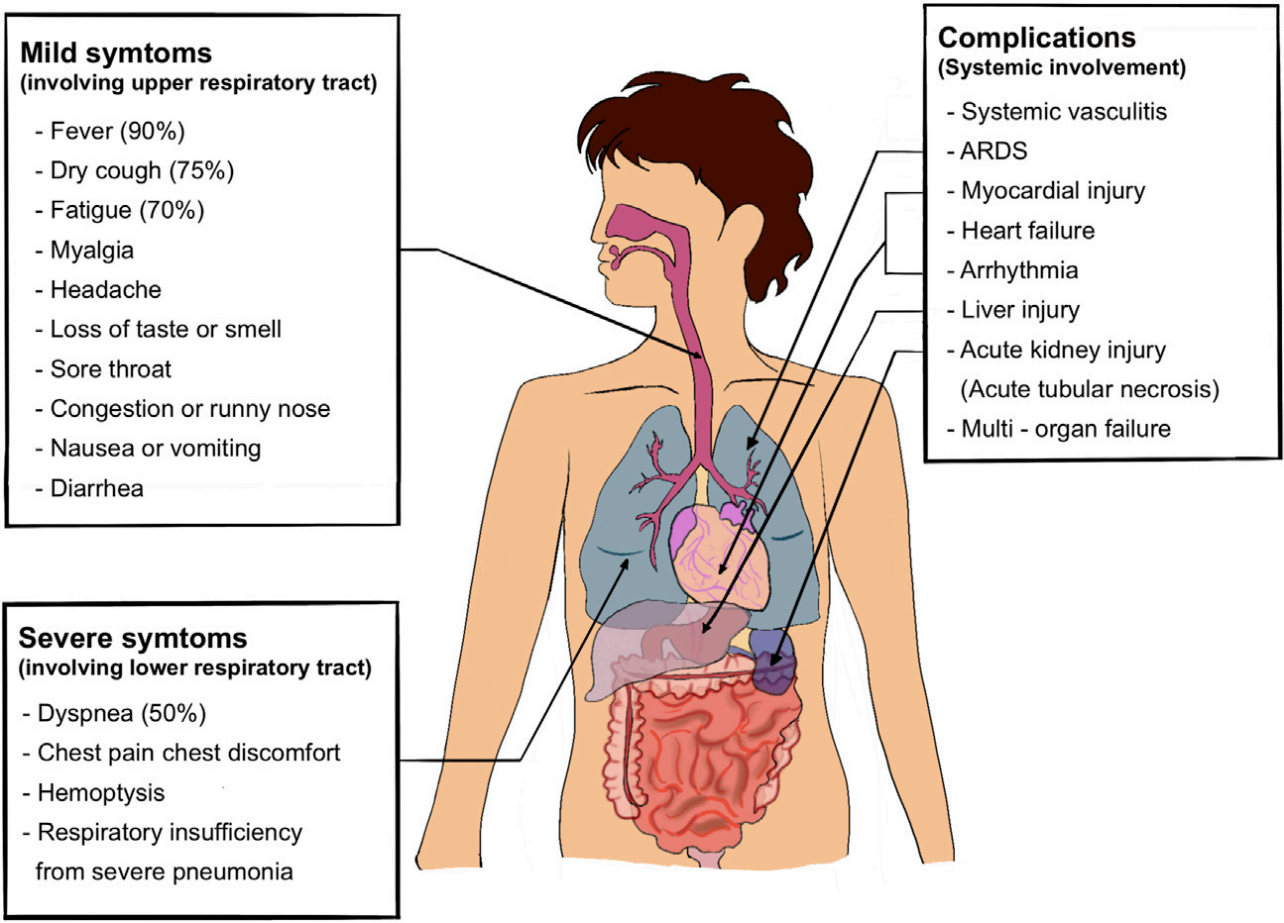
Clinical manifestation of COVID-19.

A common characteristic of SARS-CoV-2 is asymptomatic transmission, which is likely the cause of rampant spread and transmission. Given SARS-CoV-2 entry is primarily *via* the respiratory tract, upper and lower respiratory tract involvement is the most common manifestation. About one-third of patients hospitalized with SARS-CoV-2 infection meet the criteria for acute respiratory distress syndrome (Attaway et al., 2021). The main clinical manifestations of COVID-19 are fever (90% or more), cough (around 75%), and dyspnea (up to 50%) (Jiang et al., 2020). A small but significant subset has gastrointestinal symptoms. Preliminary estimates of case fatality, likely to fall as better early diagnostic efforts come into play, are about 2%, mostly due to ARDS, acute kidney injury, and myocardial injury (Jiang et al., 2020). The clinical manifestations are summarized in Figure 3 as described elsewhere (Huang et al., 2020; Jiang et al., 2020; Alizadehsani et al., 2021).

## 4 Treatments of COVID-19

It is well recognized that COVID-19 has four stages of progression in which the first stage is initiated by upper respiratory tract infection. In the second stage, the symptoms of dyspnea and pneumonia appeared. In the third stage of COVID-19, cytokine storm followed by the hyperinflammatory state predominantly worsens the clinical scenario. The final stage of COVID-19 progression is either death or recovery. While as many as 800 clinical trials are ongoing and some of these have already been completed worldwide, currently, no treatment was found to be clinically effective to act selectively against the SARS-CoV-2 infection (Becker, 2020; Stasi et al., 2020). Currently, different therapeutics are being applied to treat moderate-to-severe COVID-19 patients considering the pathological features and various stages of COVID-19, of which repurposed drugs are being used predominantly (Becker, 2020; Song et al., 2020; Stasi et al., 2020; Gavriatopoulou et al., 2021).

## 5 Repurposing Drugs for COVID-19: Concept and Mechanism of Action

Gilead Sciences first developed remdesivir (RDV) in 2017 for the treatment of infection caused by Ebola virus. In the United States, South Korea, and China, RDV was clinically evaluated in moderate-to-severe COVID-19 patients through several phase 3 clinical trials (Gavriatopoulou et al., 2021). Based on reviewing current evidence from randomized, double-blinded, placebo-controlled clinical trials, the FDA has been persuaded to believe the potential benefits of RDV over potential risks for the treatment of severe hospitalized COVID-19 patients (FDA, 2020). Henceforth, the FDA issued an Emergency Use Authorization (EUA) for emergency use of RDV for the treatment of hospitalized severe COVID-19 adult and children patients where severity of COVID-19 has been defined as SpO_2_ ≤ 94% on room air, requiring supplemental oxygen, mechanical ventilation, or extracorporeal membrane oxygenation (FDA, 2020). It is reported that RDV can inhibit RNA-dependent polymerase and may therefore be effective in the treatment of SARS-CoV-2 infection. It is actually a phosphoramidate prodrug having broad-spectrum activity against various viruses, for example, Paramyxoviridae, Filoviridae, Pneumoviridae, and Orthocoronavirinae, that is, SARS-CoV and Middle East respiratory syndrome coronavirus (MERS-CoV), as described elsewhere (Sheahan et al., 2017; Martinez, 2020; Gavriatopoulou et al., 2021). Although the FDA has recommended emergency use of RDV in severe COVID-19 patients, however, the safety and efficacy of RDV in COVID-19 patients as evidenced in multiple recent meta-analyses are controversial and inconsistent (Angamo et al., 2021; Elsawah et al., 2021; Tao et al., 2021).

Chloroquine (CQ) and hydroxychloroquine (HCQ) were included on the essential lists of medications of the World Health Organization (WHO) and used for several decades for the prophylaxis of malaria. They are also used for the treatment of rheumatoid arthritis, systemic lupus erythematosus, Sjogren’s syndrome, and post-Lyme’s disease arthritis (Shippey et al., 2018; Schrezenmeier and Dörner, 2020; Gavriatopoulou et al., 2021). Although HCQ and CQ may exhibit anti-inflammatory, immunomodulating, anti-infective, antithrombotic, and metabolic effects, however, they can also inhibit SARS-CoV-2 host entry by binding to the host cell angiotensin-converting enzyme-2 (ACE2) receptor, thereby impairing SARS-CoV-2 spike protein recognition (Becker, 2020; Fantini et al., 2020). These drugs act by blocking 2019-nCoV entry into host cells by inhibiting glycosylation of host receptors, proteolytic processing, and endosomal acidification (Sanders et al., 2020). Several clinical trials had assessed the safety and efficacy of CQ/HCQ in COVID-19 patients. Based on primary results from some clinical trials, the U.S. FDA had approved emergency use of CQ/HCQ for the treatment of COVID-19 patients on 28 March 2020. Later, the U.S. FDA issued a cautioning statement against CQ/HCQ use in COVID-19 patients due to serious cardiac toxic effects, for example, arrhythmias on April 24, 2020. Finally, on 15 June 2020, the FDA revoked the EUA use of CQ/HCQ as a potential COVID-19 therapy after accumulating negative data from clinical trials (Gavriatopoulou et al., 2021).

Lopinavir (LPV) and ritonavir (RTV) are HIV protease inhibitors and used in combination with or without other antiviral drugs for the treatment of human immunodeficiency virus (HIV-1)-infected patients older than 2 years. Due to its inhibiting nature of viral DNA-dependent RNA polymerase, either combination of LPV/RTV or alone has been recommended for the treatment of COVID-19 patients; however, the results of clinical trials are not favoring the clinical outcomes and are limited in use nowadays (Cao et al., 2020; Gavriatopoulou et al., 2021). Although LPV/RTV was suggested primarily by the National Health Commission (NHC) of China as an antiviral therapy in COVID-19 patients, however, it is not recommended by the U.S. National Institute of Health (NIH) due to lack of proven clinical effectiveness in these patients (Song et al., 2020).

Arbidol was first marketed in Russia and China as a synthetic antiviral drug for the treatment of seasonal influenza. A previous study demonstrated that arbidol was broadly effective against some other viruses including SARS-CoV and was generally well tolerated in treating these viruses (Gavriatopoulou et al., 2021). Initially, the *in vitro* test found arbidol to be an effective inhibitor of SARS-CoV-2 infection, and it was therefore recommended by the China’s NHC guide on COVID-19 treatment option. It appeared that arbidol was found ineffective against SARS-CoV-2 infection in ongoing clinical studies, although it had significant limitations in study design and sample size in these studies (Song et al., 2020).

Favipiravir was first approved in Japan for the treatment of influenza and was later found effective against Ebola virus infection also. Although several clinical trials were undertaken in China, Japan, Canada, and Russia evaluating the safety and efficacy of favipiravir alone or in combination with other antivirals against SARS-CoV-2 infection, however, the results were not persuading the clinicians for further considerations in treating COVID-19 patients with favipiravir (Gavriatopoulou et al., 2021).

Originally, darunavir/cobicistat was developed for the treatment of HIV-1 infection. Due to its protease inhibiting activity, the clinical trial had assessed the safety and efficacy of darunavir/cobicistat in SARS-CoV-2 infection and found that darunavir/cobicistat was not effective in the treatment of COVID-19 patients (Gavriatopoulou et al., 2021).

It is worth mentioning here that there are no supporting data from clinical trials that could favor the use of any HIV protease inhibitors to treat COVID-19 patients. Followingly, recently, the NIH panel for COVID-19 treatment guidelines did not recommend the use of any HIV protease inhibitors in the treatment of COVID-19 infection due to lack of clear clinical benefit in these patients (Amanat and Krammer, 2020; Gavriatopoulou et al., 2021).

Atazanavir (ATV) was discovered early in the 2000s as an antiretroviral drug for treating HIV instead of LPV/RTV because of lesser side effects of this drug. Evidence from *in silico* and *in vitro* studies suggests that by inhibiting viral major protease, ATV would inhibit SARS-CoV-2 replication even better than LPV/RTV (Fintelman-Rodrigues et al., 2020; Stasi et al., 2020; Alavian et al., 2021).

As part of highly active antiretroviral therapy (HAART), efavirenz (EVZ) and nevirapine are mainly used in the treatment of HIV/AIDS; however, these drugs could also be used for treating SARS-CoV-2 infection because of their ability to inhibit viral RNA-dependent RNA polymerase (RdRp) (Nastri, et al., 2020). Also, nelfinavir mesylate (NFV) being an antiretroviral drug may have potential efficacy against SARS-CoV-2 infection. Recent studies suggest that it can inhibit spike protein (S) medicated cell fusion of SARS-CoV-2 and may eventually block the transfer and cell-to-cell spread of SARS-CoV-2 (Yousefi et al., 2021).

Ribavirin is a nucleoside analog and was found effective against many RNA viruses, including SARS-CoV and MERS-CoV. It mainly inhibits RNA polymerase and synthesis of viral protein. Ribavirin was widely used with or without steroids against SARS infection, outbreak in 2003. Although intravenous ribavirin in combination with LPV/RTV or interferon was suggested by China’s NHC for the treatment of patients with COVID-19, it is not recommended by the NIH (Song et al., 2020).

It has well established that severe COVID-19 patients are generally associated with an increased cytokine-release syndrome, which further elevated interleukin-6 (IL-6). Tocilizumab, an IL-6 receptor antagonist, is commonly used for the treatment of rheumatoid arthritis and patients having cytokine-release syndrome (Takahashi et al., 2020). It was authorized by the Agenzia Italiana del Farmaco (AIFA), the Italian Medicines Agency, to investigate its safety and efficacy in COVID-19 patients. However, a recent clinical trial did not find significant clinical benefits, for example, reduced mortality or increased survival rate in using this drug (Stasi et al., 2020; Salama et al., 2021).

Molnupiravir, a ribonucleoside prodrug of N-hydroxycytidine (NHC), was originally developed as a potent inhibitor of respiratory syncytial virus (RSV), influenza B viruses, and influenza A viruses (IAVs) of human, avian, and swine origins (Yoon et al., 2018; Jayk Bernal et al., 2021). Later, it was found to be effective as a first oral and direct-acting anti-SARS-CoV-2 agent in both *in vitro* and *in vivo* studies (Jayk Bernal et al., 2021; Kabinger et al., 2021). When molnupiravir in the form of NHC prodrug is administered orally, it circulates systemically and is phosphorylated intracellularly to NHC triphosphate, an active form of molnupravir. This active form subsequently interferes in viral replication by inducing RNA mutagenesis through incorporation of 5′-monophosphate metabolite into viral RdRp. The active compound of molnupiravir, NHC 5′-triphosphate (NHC-TP), increases “G” to “A” and “C” to “U” transition mutations in replicating coronaviruses that lead to increased antiviral effects (Ehteshami et al., 2017; Gordon et al., 2021; Jayk Bernal et al., 2021; Kabinger et al., 2021).

Another important treatment strategy against SARS-CoV-2 infection was to develop selective targets that may neutralize monoclonal antibodies (mAbs) since previous studies reported a large number of antibodies by disturbing the receptors of either SARS-CoV or MERS coronavirus (MERS-CoV) that showed neutralization activities (Du et al., 2009; de Wit et al., 2016). Generation of virus-neutralizing antibodies or neutralizing mAbs (from the B cells of convalescent patients or humanized mice sources) are being developed from B cells of convalescent patients or humanized mice against viral infections, by targeting the receptor-binding domain (RBD) of the spike (S) protein of SARS-CoV-2 by some mechanisms—directly through triggering the phagocytosis by binding to virons or infected cells—and also by two different types of distance mechanisms in an antibody-dependent enhancement (ADE) process:

a) ADE *via* enhanced infection-expanded viral disease and replication by viral uptake into Fc gamma receptor IIa (FcγRIIa)-expressing phagocytic cells.
b) ADE *via* enhanced immune activation by excessive antibody Fc-mediated effector functions or immune complex formation in an antibody-dependent manner (Lee et al., 2020; Taylor et al., 2021).

To date, seven mAb neutralizing drugs including bamlanivimab, etesevimab, casirivimab, imdevimab, sotrovimab, cilgavimab, and tixagevimab either as monotherapy or as combination therapy have been approved or received EUAs from the U.S. FDA for the treatment of COVID-19 (Agarwal et al., 2020; Shi et al., 2020a; Zhang et al., 2021c; Nathan et al., 2021). All repurposed drugs with their mechanism of actions against SARS-CoV-2 infections are shown in Table 1.

**TABLE 1:** Possible mechanism of actions of COVID-19 therapeutics.

COVID-19 therapeutics	Potential mechanism of action against SARS-CoV-2 infection	Reference
Camostat mesilate	Protects viral entry by inhibiting TMPRSS2	Gunst et al. (2021)
Arbidol/umifenovir	Inhibits membrane fusion of the viral envelope	Yousefi et al. (2021)
Lopinavir/ritonavir/darunavir and atazanavir	Viral protease inhibitors	Alavian et al. (2021)
Remdesivir	It binds to the viral RNA-dependent RNA polymerase (RdRp), inhibiting viral replication through premature RNA transcription termination	Fricke-Galindo and Falfán-Valencia, (2021)
Favipiravir	It inhibits RdRp and synthesis of viral protein	Sanders et al. (2020)
Ribavirin	It inhibits RdRp and synthesis of viral protein	Sanders et al. (2020)
Efavirenz	Inhibits RdRp	Nastri et al. (2020)
Nevirapine	Inhibits RdRp	Nastri et al. (2020)
Tocilizumab	Reduces cytokine release by inhibiting IL-6 receptor	Takahashi et al. (2020)
Sarilumab and siltuximab	IL-6 antagonist	Yousefi et al. (2021)
Anakinra	IL-1 antagonist	Yousefi et al. (2021)
Ruxolitinib and baricitinib	Acts as an immunomodulator by inhibiting of Janus kinases and may therefore reduce the transduction of the cytokine-mediated signal	Stasi et al. (2020), Takahashi et al., (2020)
Ivermectin	Inhibits viral replication	Yousefi et al. (2021)
Oseltamivir	By inhibiting neuraminidase distributed on the surface of the virus, block viral release	Badary (2021), Yousefi et al. (2021)
Molnupiravir	Through RNA mutagenesis	Gordon et al. (2021), Kabinger et al. (2021)

**FIGURE 4:**
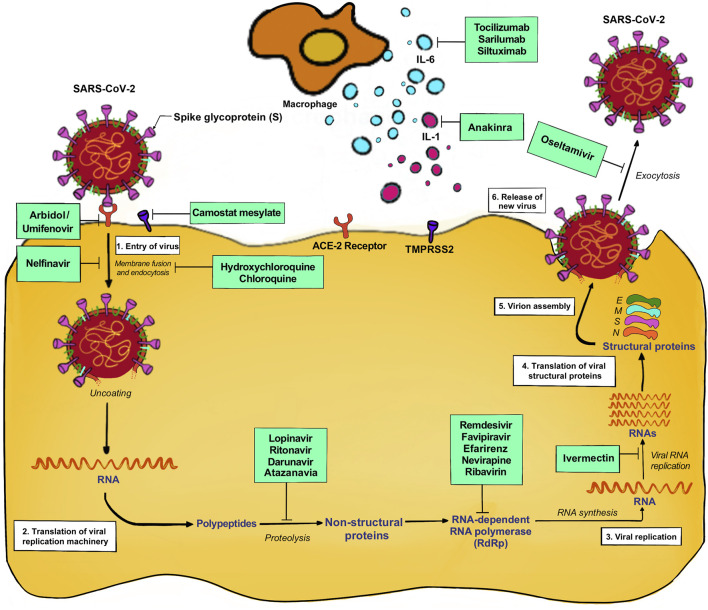
Drugs act in different phases of SARS-CoV-2 life cycle.

Also, the different drugs acting on different phases of SARS-CoV-2 life cycle are shown in Figure 4

## 6 Potential Risk of COVID-19 and Supportive Treatments

### 6.1 Anticoagulants

One of the most emerging prevalent risks associated with SARS-CoV-2 severe infection is venous thromboembolism (VTE), particularly pulmonary embolism (PE). The reported prevalence of VTE is ∼25–30% in severe COVID-19 patients, which is considerably higher than that of other viral infections (Bikdeli et al., 2020; Klok et al., 2020; Gavriatopoulou et al., 2021). Regulatory-approved drugs such as direct oral anticoagulants (DOACs), for example, rivaroxaban, apixaban, and dabigatran, and vitamin K antagonists, for example, warfarin, could be used to minimize the risk of VTE in severe COVID-19 patients. These supportive therapies should be continued for at least 3 months if VTE is suspected or confirmed in COVID-19 patients (Bikdeli et al., 2020; Gavriatopoulou et al., 2021).

### 6.2 Angiotensin-Converting Enzyme Inhibitors/Angiotensin Receptor Blockers

Initially, there was a great concern whether angiotensin-converting enzyme inhibitors (ACEIs)/angiotensin receptor blockers (ARBs) should or should not be continued in COVID-19 patients, especially with hypertension and diabetes mellitus. This is because SARS-CoV-2 binds to the ACE2 receptor to gain entry into the host cells. However, recent meta-analyses established that COVID-19 patients taking ACEIs/ARBs were not associated with an increased risk of mortality compared to those not taking ACEIs/ARBs. The risk of composite severe clinical manifestations was not significantly different between the positive patients with or without ACEI/ARB users and also found evidence of beneficial effects for using ACEIs/ARBs especially in hypertensive COVID-19 patients. These results strongly suggest continuing with renin angiotensin aldosterone system (RAAS) inhibitors during the COVID-19 pandemic (Baral et al., 2020; Biswas and Kali, 2021a; Wang et al., 2021).

### 6.3 Antiplatelets

A recent meta-analysis indicated that the risk of acute stroke was significantly higher in severe COVID-19 patients than in non-severe COVID-19 patients (RR = 4.18, 95% CI: 1.7–10.25; *p* = 0.002) (Siepmann et al., 2021). Clinical studies also showed that heart failure/myocardial infarction (MI) is prevalently higher in severe COVID-19 patients. The P2Y12 receptor antagonists, for example, clopidogrel, prasugrel, and ticagrelor, are widely used as first-line therapy in patients with stroke or coronary artery disease (CAD) (Bikdeli et al., 2020; Sivaloganathan et al., 2020; Zhao et al., 2021).

### 6.4 Antifibrotics

Idiopathic pulmonary fibrosis is one of the major risk factors associated with the severity of COVID-19. Magnitude and intensity of lung fibrosis may increase the risk for severe clinical outcomes in patients with COVID-19. It is proposed that antifibrotics such as pirfenidone and nintedanib may reduce the severity of SARS-CoV-2 infection and might be an integral part of COVID-19 therapeutics (Bikdeli et al., 2020; Gavriatopoulou et al., 2021).

### 6.5 Systemic Corticosteroids

During severe acute respiratory syndrome coronavirus (SARS-CoV) outbreak in 2002–2004, steroid therapy was commonly administered along with other medications. Initially, the WHO did not support their use without the results of clinical trials being assessed and only recommended their strict use in especial clinical circumstances. However, with the progression of the pandemic, robust evidence for the associations of corticosteroids with the clinical outcomes in COVID-19 is becoming available since steroids are being currently administered in many parts of the world (Mattos-Silva et al., 2020).

A recent retrospective study indicated that methylprednisolone was associated with a decreased risk of death (HR 0.38; 95% CI 0.20–0.72) in patients with severe COVID-19, who developed ARDS (Wu et al., 2020). Another retrospective study revealed that COVID-19-hospitalized patients taking steroids were associated with a significantly lower mortality rate than those who did not take steroids (13.9 vs. 23.9%; HR 0.51, 95% CI 0.27–0.96, *p* = 0.044) (Fernández-Cruz et al., 2020). Very recently, an open-label randomized controlled trial (RCT) showed that COVID-19-hospitalized patients taking dexamethasone were associated with a significantly lower rates of 28-day mortality than the patients taking standard of care (RR 0.83, 95% CI 0.74–0.92, *p* = 0.0007). This study further revealed that dexamethasone reduced mortality significantly in ventilated COVID-19 patients (RR 0.65, 95% CI 0.48–0.88, *p* = 0.0003) as well as in patients who have taken supplemental oxygen (RR 0.80, 95% CI 0.67–0.96, *p* = 0.0021) (Horby et al., 2021).

Systemic steroids especially dexamethasone in specific COVID-19 patients, for example, critically ill or require supplemental oxygen, may be considered based on the current available evidence. The clinical benefits of dexamethasone use may be apparent in COVID-19 patients if they were treated for greater than 7 days after the onset of COVID-19-related symptoms (Gavriatopoulou et al., 2021).

### 6.6 Bronchodilators/Vasodilators

Bronchodilators may be administered whenever indicated in COVID-19 patients. Severe COVID-19 patients with hypoxemia may be particularly benefited from the pulmonary vasodilators. Although the lack of rigorous evidence did not favor the use of pulmonary vasodilators, for example, nitric oxide in COVID-19 patients, a recent, open-label, parallel-group, phase 2, RCT indicated that early inhalation of budesonide reduced the risk of urgent medical care support and also reduced the time to recover from early COVID-19 diagnosis (Gavriatopoulou et al., 2021; Ramakrishnan et al., 2021).

### 6.7 Non-Steroidal Anti-Inflammatory Drugs

Since fever and pain are common in SARS-CoV-2 infection, paracetamol should be generally considered as a first-line antipyretic and analgesic agent if not contraindicated due to other clinical conditions. However, ibuprofen may be reserved for patients who are unable to tolerate paracetamol until further studies clarify the adverse and beneficial effects of non-steroidal anti-inflammatory drugs (NSAIDs) in patients with COVID-19 (Robb et al., 2020; Gavriatopoulou et al., 2021).

## 7 Pharmacogenomics and Precision Medicine for COVID-19

Pharmacogenomic considerations of currently used COVID-19 therapeutics may help clinicians to optimize the efficacy or safety of these drugs, and may accelerate the development of precision COVID-19 medicine. To mitigate devastating catastrophe associated with COVID-19, many drugs without establishing robust evidence of efficacy or magnitude of toxicities have been used in these patients either as an off-label/compassionate use or as a clinical trial under these unprecedented health situations as an urgent attempt. Although pharmacogenomic determinants are very important considerations in optimizing the efficacy or toxicity of many repurposed antiviral drugs or supportive treatments, they are the most neglected issues in COVID-19 therapeutics since there are almost no pharmacogenomics data for these drugs (Takahashi et al., 2020). It is very unlikely that pharmacogenomic associations of COVID-19 therapeutics with the efficacy or safety have not been considered yet in any clinical studies.

## 8 Pharmacogenomic Landscape of COVID-19 Therapies

### 8.1 Genetic Variants Affecting the Safety or Efficacy of COVID-19 Therapies

#### 8.1.1 Drug-Metabolizing Enzymes

Many drugs either repurposed antivirals or supporting medications that are used in the treatment of COVID-19 are metabolized by a number of drug-metabolizing enzymes called cytochrome P450 (CYP) enzymes. Genetic variants of the *CYP* genes encoding these important CYP enzymes may regulate their expression and may also contribute to drug response variability by altering the PK properties of the respective drugs. Therefore, for achieving optimal efficacy or safety of COVID-19 therapeutics, *CYP* genes of interest should be considered in future clinical studies to investigate such genetic associations. Here, we will summarize all *CYP* genes with potential interest of single-nucleotide polymorphisms (SNPs) as well as other genes affecting the PK profile of COVID-19 therapeutics with potential considerations of pharmacogenomics (PGx) of these drugs in other clinical conditions as evidenced from the literature (Table 2).

**TABLE 2:** Evidence of pharmacogenetic associations of COVID-19 therapies in other disease conditions.

Drug	Gene	SNP	Effects on PK/safety/efficacy	MAF using 1,000 genome database	Disease	Reference
Atazanavir	*SLCO1B1*	rs4149056	Enhanced toxicity	C = 8.8%	HIV	Bonora et al. (2015)
	*ABCB1*	rs2032582	Hyperbilirubinemia	A = 33.4%	HIV	Rodríguez-Nóvoa et al. (2007)
	*UGT1A1*	rs8175347	Hyperbilirubinemia	AT = 34.8%	HIV	Rodríguez-Nóvoa et al. (2007), Du et al. (2019)
	*CYP3A5*	rs2740574	Efficacy	C = 23.1%	HIV	Cafiero et al. (2020)
Azithromycin	*ABCB1*	rs1045642	Variability in AUC	A = 39.5%	Healthy volunteers	He et al. (2009)
Clopidogrel	*CYP2C19*	rs4244285	Efficacy	A = 22.1%	ACS	Scott et al. (2013)
Dexamethasone	*UGT1A1*	rs4148323	Efficacy	A = 3.4%	Cancer	Yamasaki et al. (2015)
	*ABCB1*	rs1045642	Efficacy	A = 39.5%	Autoimmune disease	Song et al. (2017)
Darunavir	*SLCO3A1*	rs8027174	PK	T = 4.7%	HIV	Moltó et al. (2013)
Efavirenz	*CYP2B6*	rs3745274	CNS toxicity, suicide attempt	T = 31.6%	HIV	Sukasem et al. (2012), McDonagh et al. (2015), Desta et al. (2019)
	*ABCB1*	rs3842	PK	C = 18.8%	Healthy volunteers	Mukonzo et al. (2009)
Ivermectin	*CYP3A4*	rs35599367	PK	A = 1.5%	Healthy volunteers	González Canga et al. (2008)
	*ABCB1*	rs1045642	PK	A = 39.5%	A549 cell lines	Lespine et al. (2006)
Lopinavir	*SLCO1B3*	rs717620	Dyslipidemia and hyperbilirubinemia	T = 13.5%	HIV	da Rocha et al. (2015)
	*ABCC2*	rs8187710	Dyslipidemia and hyperbilirubinemia	A = 6.8%	HIV	Lubomirov et al. (2010)
	*SLCO1B1*	rs4149056	Efficacy	C = 8.8%	HIV	Lubomirov et al. (2010)
	*ABCB1*	rs1045642	Safety/efficacy	A = 39.5%	HIV	Rakhmanina et al. (2011)
	*CYP3A5*	rs2740574	Efficacy	C = 23.1%	HIV	Rakhmanina et al. (2011)
Losartan	*CYP2C9*	*rs1799853*	PK	T = 4.8%	Cell lines	Iwamura et al. (2011)
	*ABCB1*	rs1045642	Efficacy	A = 39.5%	Hypertension	Göktaş et al. (2016)
Ribavirin	*SLC28A2*	rs11854484	Anemia	T = 30.3%	HCV	Allegra et al. (2015)
Ritonavir	*ABCB1*	rs1045642	PK	A = 39.5%	HIV	Rakhmanina et al. (2011)
	*CYP3A5*	rs2740574	Efficacy	C = 23.1%	HIV	Rakhmanina et al. (2011)
	*SLCO1B1*	rs4149056	Efficacy	C = 8.8%	HIV	Lubomirov et al. (2010)
Umifenovir	*CYP3A4*	rs35599367	PK	A = 1.5%	Healthy volunteers	Deng et al. (2013)
Warfarin	*CYP2C9*	rs1799853	Toxicity	T = 4.8%	Thromboembolism	Johnson et al. (2017)

Here, SNP, single-nucleotide polymorphism; PK, pharmacokinetic; MAF, Minor allele frequency; AUC, area under concentration; SLE, systemic lupus erythematosus; DLE, discoid lupus erythematosus; ACS, acute coronary syndrome.

It is important to recognize that *CYP* genetic variants are highly polymorphic and only few of these genetic variants are associated with the safety or efficacy of the respective drugs. The most prevalent and studied genetic variants of the *CYP3A4/5*, *CYP2B6*, *CYP2C8*, *CYP2C9*, *CYP2C19*, and *CYP2D6* pharmacogenes have wide inter-ethnic variability. For example, CYP2D6*4 is highly prevalent in American population, whereas CYP2C19*2, *3 is highly prevalent in South Asians (Sukasem et al., 2013; Medhasi et al., 2016; Zhou et al., 2017; Biswas, 2021a; Sukprasong et al., 2021).

#### 8.1.2 Transporters

In addition to *CYP* genes, some other efflux or uptake transporter proteins encoded by the transporter genes may also modify the PK properties of COVID-19 therapeutics and may be associated with the efficacy or safety variability. Like *CYP* genetic variants, transporter genes such as *ABCB1*, *SLCOIB1*, and *ABCC2* also have interindividual variabilities and may affect the safety or efficacy of drugs accordingly (Sensorn et al., 2013; Medhasi et al., 2016; Atasilp et al., 2020; Biswas, 2021b). The list of transporter genes with relevant COVID-19 drugs with potential considerations of PGx in other clinical conditions as evidenced from the literature is shown in Table 2.

### 8.2 Other Genes Affecting the Severity of SARS-CoV-2 Infection

#### 8.2.1 *HLA*

The human leukocyte antigen (HLA) encoded by the *HLA* gene is located on chromosome 6p21 which contains crucial regulators of immune response. The classical genes *HLA-A*, *HLA-B*, and *HLA-C* are in Class I and the classical genes *HLA-DP*, *HLA-DQ*, and *HLA-DR* are in Class II (Wang et al., 2015). HLA Class I has a role to present pathogen peptides to CD8^+^ T cell, becoming cytotoxic T cell which can directly destroy infected cell by inducing apoptosis (cellular immunity), whereas HLA class II has a role to present pathogen peptides to CD4^+^ T cell, activating B cell to become plasma cell and produce antibodies (humoral immunity) (Figure 1). However, in the case of viral infection such as COVID-19, cellular immunity is more important than humoral immunity to clear out the viruses which are staying inside the host cells (Le Bert et al., 2020).

Some variation of *HLA* alleles also has an association with some certain disease. The recently introduced genome-wide association study (GWAS) has suggested that several genes converging in common pathways contribute to the genetic susceptibility in several disorders, such as ankylosing spondylitis, psoriasis, chronic beryllium disease, rheumatoid arthritis, celiac disease, type 1 diabetes, and multiple sclerosis (Fiorillo et al., 2017). For example, 90–95% of patients with ankylosing spondylitis have *HLA-B27* (Zhu et al., 2019). *HLA* alleles are not only associated with a set of autoimmune disease; the study in Thai children with autism showed that HLA-B*13:02, HLA-B*38:02, HLA-B*44:03, and HLA-B*56:01 alleles were significantly increased in autistic subjects compared with normal subjects (Puangpetch et al., 2015).

In addition to an association with some diseases, *HLA* alleles are also associated with an increased risk of certain drug allergy (Sukasem et al., 2014b; 2018b). The study in Thai population shows that HLA-B*15:02 is strongly associated with aromatic antiepileptic drug (AED)-induced Stevens-Johnson syndrome (SJS)/toxic epidermal necrolysis (TEN) (Sukasem et al., 2021b); HLA-B*15:02 and HLA-C*08:01 alleles are significantly associated with co-trimoxazole (CTX)-induced SJS/TEN, whereas the HLA-B*13:01 allele was significantly associated with CTX-induced drug reaction with eosinophilia and systemic symptoms (DRESS) (Sukasem et al., 2020a); HLA-B*46:01, HLA-B*56:02/04, and HLA-B*40:01 alleles contribute to the risk of phenytoin-induced cutaneous adverse drug reactions (PHT-induced cADRs) (Sukasem et al., 2020b); carbamazepine-induced SJS/TEN shows an association with *HLA-B∗15:21* allele (Jaruthamsophon et al., 2017; Sukasem et al., 2018a); *HLA-A* ^*∗*^ *02:07* and *HLA-B* ^*∗*^ *15:02* are associated with lamotrigine (LTG)-induced cutaneous adverse drug reactions (cADRs) (Koomdee et al., 2017); HLA-B*13:01 is associated with dapsone-induced severe cutaneous adverse reactions including SJS/TEN and DRESS (Tempark et al., 2017); and HLA-B*58:01 is associated with allopurinol-induced SJS/TEN, and screening for HLA-B*58:01 alleles in patients who will be treated with allopurinol would be clinically helpful in preventing the risk of developing cADRs (Sukasem et al., 2016b). The future of pharmacogenomics-guided therapy in clinical settings across Thailand appears promising because of the availability of evidence of clinical validity of the pharmacogenomics testing (Sukasem et al., 2021a). The effectiveness of *HLA* screening on a wider scale in clinical practice requires significant resources, including state-of-the-art laboratory; multidisciplinary team approach, and healthcare provider education and engagement; clinical decision support alert system *via* electronic medical record (EMR); laboratory standards and quality assurance; evidence of cost-effectiveness; and cost of pharmacogenomic tests and reimbursement (Jantararoungtong et al., 2021a).

The severity of COVID-19 ranges from being asymptomatic to developing into a fatal acute respiratory syndrome and varies between populations. This can be linked to the variations in the *HLA*. The set of *HLA* alleles might determine the immune responses to viruses according to the selected peptides that can bind to the peptide-binding groove. A recent study from Italy showed an increase in the frequency of HLA-B*27:07, HLA-DRB1*15:01, and HLA-DQB1*06:02 among severe COVID-19 patients in a cohort of 99 Italians (Novelli et al., 2020). However, another study from Sardinia in Italy showed a negative influence on the disease course in the presence of the HLA-DRB1*08:01 allele (Littera et al., 2020). A study from Spain showed that the HLA-A*11, HLA-C*01, and HLA-DQB1*04 were associated with higher mortality in a cohort of 72 patients (Lorente et al., 2021). A study from 95 South Asian patients showed an increase in the frequency of HLA-B*51 and HLA-DRB1*13 in the fatal group compared to the mild infection group, while increase in the frequency of HLA-B*35 among the mildly infected group (Naemi et al., 2021). A study in 147 individuals of European descent experiencing variable clinical outcomes following COVID-19 infection showed that a significant difference in the allele frequency of HLA-DRB1*04:01 was found in the severe patient compared to the asymptomatic group, whereas a significantly lower frequency of the HLA-DQA1*01:01, HLA-DQB1*05:01, and HLA-DRB1*01:01 alleles was found in the asymptomatic group compared to the background population (Langton et al., 2021). A discrepancy between the studies can be attributed to many factors, including sample size and ethnic variations.

In addition to the prediction of certain diseases and certain adverse drug effects, an association between polymorphism in the *HLA* system and COVID-19 severity might have an impact on the implementation of a screening program to identify individuals at risk for COVID-19. In Thailand, the top ranked *HLA* alleles include HLA-A*11:01 (26.06%), HLA-B*46:01 (14.04%), HLA-C*01:02 (17.13%), HLA-DRB1*12:02 (15.32%), HLA-DQA1*01:01 (24.89%), and HLA-DQB1*05:02 (21.28%), and when focusing on *HLA-B*, the most frequent alleles were HLA-B*46:01 (11.51%), HLA-B*58:01 (8.62%), HLA-B*40:01 (8.22%), HLA-B*15:02 (8.16%), HLA-B*13:01 (6.95%), and HLA-B*44:03 (4.21%) (Puangpetch et al., 2014; Satapornpong et al., 2020). According to the study from Spain, Thai people should be alert because HLA-A*11and HLA-C*01 alleles are associated with high mortality. Moreover, Thai population has less frequency of a good prognostic marker such as HLA-B*35 studied from South Asia. However, clinical analysis of the association between *HLA* allele frequency and COVID-19 severity in Thailand is needed to validate the *HLA* alleles as the appropriate prognostic markers used in Thai clinical practice.

#### 8.2.2 *ACE2*

Angiotensin-converting enzyme-2 (ACE2) is a protein consisting of 805 amino acids encoded by the *ACE2* gene and is expressed in many parts of human cells including oral mucosa and nasopharynx. It is well recognized that ACE2 serves as an entry binding receptor of SARS-CoV-2 through interactions with specific amino acids of this enzyme (Sanders et al., 2020). A recent *in silico* model of possible *ACE2* genetic variants with its interaction with the SARS-CoV-2 spike (S) protein has been analyzed, and it revealed that both rs73635825 (S19P) and rs143936283 (E329G) were shown to interfere with the ACE2 interaction with the S protein of SARS-CoV-2 (Ambrocio-Ortiz et al., 2021). After analyzing SNPs of *ACE2* with susceptibility to SARS-CoV-1 or MERS, recent studies predicted that certain SNPs of *ACE2* should consider COVID-19 patients for assessing the correlation with severity. It was also predicted that COVID-19 severity would vary around the world since the prevalence of the *ACE2* genetic variants was significantly different in various ethnic groups (Benetti et al., 2020; Gemmati and Tisato, 2020; Ambrocio-Ortiz et al., 2021; Bakhshandeh et al., 2021; Biswas, 2021c; Choudhary et al., 2021).

#### 8.2.3 *TMPRSS2*

Transmembrane protease, serine 2 (TMPRSS2) is an enzyme of serine protease family encoded by the *TMPRSS2* gene. During membrane fusion, SARS-CoV-2 “S” protein is activated by the TMPRSS2; therefore, it is postulated that *TMPRSS2* variants might have been correlated to COVID-19 severity. Genetic variants of *TMPRSS2* augmenting TMPRSS2 activity might play an important role in the progression of COVID-19 severity and may be considered as a genetic risk factor (Hou et al., 2020; Choudhary et al., 2021).

## 9 Pharmacogenetics Considerations of COVID-19 Therapeutics: Implications for Efficacy and Safety

### 9.1 Antiparasitics

Due to proven ineffectiveness and exclusion from COVID-19 treatment protocols, we have excluded hydroxychloroquine and chloroquine from further analysis in this review.

#### 9.1.1 Ivermectin

Ivermectin underwent extensive metabolism *via* CYP enzymes, predominantly by the CYP3A4 isoform, converting ivermectin into at least 10 metabolites, most of which are hydroxylated and demethylated products (Zeng et al., 1998; González Canga et al., 2008). Ivermectin is also a substrate of P-gp encoded by the *ABCB1*, and genetic polymorphisms of *ABCB1* have linked to severe neurologic ADRs (Lespine et al., 2006; Baudou et al., 2020). Also, ivermectin is transported by the OATP1A2 and OATP2B1 encoded by the *SLCO1A2* and *SLCO2B1*, respectively, although no pharmacogenetic study was identified for this association to date in the literature (Fricke-Galindo and Falfán-Valencia, 2021; Telbisz et al., 2021).

### 9.2 Antiviral Drugs

#### 9.2.1 Remdesivir

Second, although remdesivir (RDV) is a promising investigational drug proving its activity in cell culture and animal models against SARS-CoV, Middle East respiratory syndrome corona virus (MERS-CoV), and SARS-CoV-2, it is currently not approved for any indication (FDA, 2020). *In vitro* studies suggest that RDV is a substrate for multiple drug metabolizing enzymes, for example, CYP2C8, CYP2D6, and CYP3A4, and also a substrate of OATP1B1 and P-glycoprotein (P-gp) transporters (Takahashi et al., 2020; Deb et al., 2021). Although the pharmacogenomic study of RDV has not been undertaken yet, it is predicted that known variants of these metabolic/transporter genes could affect the safety or efficacy of remdesivir and should assess COVID-19 patients. It is important to note that all of these genes were considered very important pharmacogenes (VIPs) by PharmGKB (Takahashi et al., 2020).

In addition to CYP/transporters’ involvement in the PK, RDV prodrug undergoes an intra-cellular sequential metabolism predominantly mediated by hydrolase activity to an active C-adenosine nucleoside triphosphate analog (Eastman et al., 2020; European Medicines Agency, 2020). Upon diffusion of RDV into the cell, the conversion of RDV into the nucleoside monophosphate form is presumably initiated by carboxylesterase (CES)-mediated hydrolysis of the amino acid ester that liberates a carboxylate and then converted to cyclic anhydride (Eastman et al., 2020; McCreary and Pogue, 2020; Yang, 2020). Cyclic anhydride is very unstable and hydrolyzed by water to the alanine metabolite GS-704277 which is further hydrolyzed by phosphoramidase to the nucleoside monophosphate. Nucleoside monophosphate is further phosphorylated in the presence of nucleoside phosphate kinase enzyme, yielding the active nucleoside triphosphate analog which may act as an analog of adenosine triphosphate (ATP) and competes with the natural ATP substrate to selectively inhibit RNA-dependent RNA polymerase (RdRp). Since the conversion of RDV to pharmacologically active nucleoside triphosphate analog is initiated extensively by the intracellular CES, we predict that genetic variability of *CES* gene-regulating expression may affect the safety or efficacy of RDV, and it is therefore hypothesized that “COVID-19 patients inheriting *CES* genetic polymorphisms might potentially modify efficacy or safety of RDV warranting clinical studies to be assessed for the achievement of precision medicine of RDV.”

#### 9.2.2 Ribavirin

A genetic association study of ribavirin in some other viral infections except SARS-CoV-2 revealed that some genetic polymorphisms may result in up to 30% variability of ribavirin trough concentrations, affecting its safety and efficacy. It was found that patients who carried the homozygous variant of the *SLC29A1*, encoding influx transporter, were associated with significantly higher trough concentrations than wild-type variants (2,070 ng/ml vs. 1,837 ng/ml; *p* = 0.02). By contrast, patients who carried the homozygous variant of the *SLC28A2* were associated with significantly lower trough concentrations than wild types (homozygous 1,595 ng/ml vs. heterozygous 1,933 ng/ml vs. wild-type 2,229 ng/ml; *p* = 0.04). This is also consistent with *SLC28A3* variant (homozygous variant 2,294 ng/ml vs. heterozygous variant 1,813 ng/ml; *p* = 0.01) (Allegra et al., 2015). It is also well-established that hemolytic anemia, the most common dose-limiting toxic effect of ribavirin, is protected by various genetic variants of *ITPA*, encoding inosine triphosphatase (D’Avolio et al., 2016). In a meta-analysis of 20 studies, hemoglobin was significantly reduced in patients with wild-type alleles of ITPA compared to the patients having single-nucleotide polymorphisms (SNPs) as reported in a meta-analysis consisting of 20 studies (OR: 12.8, 95% CI: 7.4–22.1 for rs1127354 CC; OR: 3.4, 95% CI: 2.1–5.6 for rs7270101 AA; OR: 4.4, 95% CI: 2.8–7.0 for rs6051702 AA) (Pineda-Tenor et al., 2015). It was established that reduced activity of *ITPA* due to genetic variants governs to the deposition of inosine triphosphate and safeguard against ribavirin-induced very toxic effects, that is, hemolysis. Hemolytic anemia was also found for a short-term use of ribavirin in respiratory viral infection (Burrows et al., 2015). By contrast, a genome-wide association study (GWAS) of 303 patients with hepatitis C viral infection who were administered ribavirin with other therapy showed that the risk of thrombocytopenia was significantly higher in patients with rs6139030 SNP of *ITPA* (OR: 3.9, 95% CI: 2.8–5.5, *p* = 1.33 × 10–15) (Tanaka et al., 2011). Approximately 25% of the Thai patients carried *ITPA* genetic polymorphisms (Jantararoungtong et al., 2021b), which revealed that considerable proportion of Thai population taking ribavirin would be affected by the *ITPA* genetic variabilitites.

While the association of influx transporter genetic variants or *ITPA* with the safety or efficacy of ribavirin was not investigated in COVID-19 patients, such genetic association assessments are warranted in future clinical studies.

#### 9.2.3 Favipiravir

Favipiravir (FPV) is one of the most potential antiviral drugs currently under considerations in several clinical trials to evaluate its efficacy and safety in patients with COVID-19 (Du and Chen, 2020). The National Medical Products Administration (NMDA) of China approved emergency use of FPV for a clinical trial in adult patients with COVID-19. Being a prodrug, FPV is ribosylated and phosphorylated to form active metabolite called FPV ibofuranosyl-5′-triphosphate, which then competes with purine nucleosides and interferes the viral replication by potentially inhibiting the RdRp of RNA viruses, for example, SARS-CoV-2 (Du and Chen, 2020). FPV is metabolized mainly by aldehyde oxidase and to a less extent by the xanthine oxidase (Takahashi et al., 2020). Although there are no published studies that have specifically assessed the pharmacogenomic influence of FPV, genetic variants of aldehyde oxidase were associated with pharmacodynamic outcomes in other drugs which are substrates of aldehyde oxidase such as azathioprine or allopurinol, and suggesting that PGx of FPV should also be taken into considerations in COVID-19 patients (Takahashi et al., 2020).

#### 9.2.4 Oseltamivir

Oseltamivir is a prodrug which is converted to the active metabolite *via* carboxylesterase 1 (CES1) encoded by the *CES1* (Shi et al., 2006). The SNP rs71647871 of *CES1* has been found to be associated with variation in plasma concentration–time curve of oseltamivir (Tarkiainen et al., 2012) The PK of oseltamivir may also be affected by the *ABCB1*, *CES1*, *NEU2*, and *SLC15A1* genetic variants. The SNP rs1045642 of *ABCB1* was associated with neurologic ADRs developed by oseltamivir (Bermúdez de León et al., 2020; Fricke-Galindo and Falfán-Valencia, 2021).

#### 9.2.5 Nevirapine

Certain genetic polymorphisms of *HLA* and *CYP2B6* may be associated with increased risk of SJS/TEN when treated with nevirapine as reported elsewhere (Martin et al., 2005; Badary, 2021). Also, selective *ABCB1* genetic variants may also be responsible for developing hepatotoxicity as evidenced elsewhere when treated with nevirapine (Vitezica et al., 2008; Badary, 2021).

### 9.3 Antiretroviral Agents

#### 9.3.1 Lopinavir/Ritonavir

After pharmacogenomic analysis of 1,380 variants in 638 HIV-infected Caucasian patients taking LPV/RTV, four significant variants were identified. LPV/RTV clearance was higher in patients who carried SLCO1B1*4/*4 homozygous variants and was lower in patients who carried two or more variant alleles of the SLCO1B1*5, *ABCC2*, or *CYP3A* tag than in the patients of the reference group (Lubomirov et al., 2010). GWAS after analyzing 290 variants with the toxicity of LPV/RTV among 104 Caucasian patients with HIV revealed that dyslipidemia and hyperbilirubinemia were significantly associated with some genetic variants of the *CETP*, *MCP-1*, *ABCC2*, *LEP*, and *SLCO1B3* genes. Also, a genetic variant of the *IL-6* gene was significantly associated with resulting in diarrhea (all *p* < 0.01) (Aspiroz et al., 2014; Takahashi et al., 2020).

In addition to these, LPV/RTV being a substrate of P-gp was encoded by the *ABCB1* gene. The efficacy and safety of these drug combinations may also be affected by the genetic polymorphisms of *ABCB1* encoding P-gp expression. Over 30% of the Thai patients inherited *C3435T ABCB1* genetic polymorphisms (Sensorn et al., 2013, 2016), suggesting that considerable proportion of Thai population be affected by the *C3435T ABCB1* genetic variant if taking LPV/RTV for combating COVID-19. A recent review hypothesized that the safety or efficacy of LPV/RTV may be affected by the *C3435T* SNP of *ABCB1*, and the risk phenotypes due to carrying this SNP were prevalently highest in Europe (76.8%), followed by America (67%), Asia (63.5%), and Africa (41.4%) (Biswas, 2021b).

#### 9.3.2 Darunavir/Cobicistat

Darunavir being a substrate of CYP3A4 was used simultaneously with cobicistat, a CYP3A4 inhibitor in a clinical trial for COVID-19 for increasing the exposure of darunavir (Takahashi et al., 2020). Genetic variants of *CYP3A4* regulating the function or expression of CYP3A4 may affect the safety or efficacy of darunavir/cobicistat in COVID-19 patients and should be considered in future studies (Takahashi et al., 2020). Although there is no direct evidence that darunavir is a substrate of SLCO3A1, a 12% significantly lower Darunavir clearance was reduced in patients with *SLCO3A1* variant, suggesting that this might be a substrate of darunavir and should assess COVID-19 patients (*p* < 0.05) (Moltó et al., 2013).

#### 9.3.3 Atazanavir

Atazanavir (ATV) is metabolized by UGT1A and is also an inhibitor of CYP3A. Several genetic polymorphisms of *UGT1A1*, for example, UGT1A1*6, *28, *36, *37, and *80, may affect the PK of ATV and may produce toxicity as outlined in the CPIC dosing guidelines. The CPIC pharmacogenomic-based dosing guidelines have recommended to counseling the patients carrying these variants because of possibility for developing severe hyperbilirubinemia (Gammal et al., 2016). A rapid, reliable, cost-effective, and simple assay to detect *UGT1A1* genetic polymorphisms in has already been developed for adoption in routine clinical practice (Sukasem et al., 2016a). The metabolism of ATV is also partially governed by the P-gp encoded by the *ABCB1*, and patients carrying *C3435T ABCB1* SNP may be at risk of hyperbilirubinemia and severe jaundice as well. Numerous studies showed that certain genetic polymorphisms of *APOA5*, *APOC3*, *ABCA1*, and *APOE* genes were associated with increased risk of dyslipidemia in patients taking atazanavir (Zanone Poma et al., 2008; Suwalak et al., 2015; Badary, 2021).

#### 9.3.4 Efavirenz

Since efavirenz is predominantly detoxified by the CYP2B6, therefore, patients may be at increased risk for toxicity such as depression and suicidal tendencies with some *CYP2B6* genetic variants, reducing the function of CYP2B6 (McDonagh et al., 2015; Desta et al., 2019). Pharmacogenomics for this drug have been extensively studied including in Thai HIV patients, and the CPIC guideline has already been developed for guiding patients with CYP2B6 genetic variants (Sukasem et al., 2012; Sukasem et al., 2014a; Manosuthi et al., 2014; Desta et al., 2019). The SNP rs4803419 of *CYP2B6* was independently associated with increased plasma efavirenz concentration as found in a GWAS (Holzinger et al., 2012). Serious toxic effects of efavirenz, for example, depression and suicidal tendencies, can be optimized by adjusting the dose based on *CYP2B6* genotyping results of patients (Desta et al., 2019).

### 9.4 Interferon β-1b

An interferon (INF) regulatory factor (IRF6) encoded by the *IRF6* was significantly associated with increased risk of liver injury as identified in a case–control study of IFN-β1b-treated multiple sclerosis patients (OR: 8.3, 95% CI: 3.6–19.2; *p* = 2.3 × 10^–8^). The results were subsequently confirmed in an independent cohort study of patients with multiple sclerosis in which liver injury was proved with significantly increased aspartate aminotransferase and alkaline phosphatase concentrations for those who carried *IRF6* genetic variants (Kowalec et al., 2018; Takahashi et al., 2020).

### 9.5 IL-6 and IL-1 Antagonists

Genetic polymorphisms of the *FCGR3A*, *IL6R*, *CD69*, and *GALNT18* genes may affect the efficacy of tocilizumab in RA as reported elsewhere (Maldonado-Montoro et al., 2016; Maldonado-Montoro et al., 2018; Jiménez Morales et al., 2019). It was reported that the *FCGR3A* rs396991TT genotype had a higher response rate at 12 months therapy of tocilizumab in 87 patients with RA (OR: 5.1; 95% CI: 1.2–21.3; *p* = 0.03). Specific Fc fragment of the IgG receptor binding to tocilizumab may be altered by this selective genetic variant and may change systemic clearance of this drug (Jiménez Morales et al., 2019). Polymorphisms of other genes, for example, *IL6R*, *CD69*, and *GALNT18*, have limited direct effects on the safety or efficacy of tocilizumab (Maldonado-Montoro et al., 2016, 2018). Also, relevant pharmacogenomic data affecting either safety or efficacy of other IL-6 or IL-1 antagonists, that is, sarilumab, siltuximab, and anakinra, were not found in the literature (Takahashi et al., 2020). Although considerations of all of these pharmacogene are highly speculative, at least *FCGR3A* rs396991TT SNP should be replicated in COVID-19 patients.

### 9.6 Inhibitors of the Renin Angiotensin Aldosterone System

Renin angiotensin aldosterone system (RAAS) inhibitors are affected by the *CYP2C9* and *ABCB1* genetic variabilities. For example, patients carrying reduced function alleles of *CYP2C9*, that is, *2, *3, may develop toxicity if taking losartan and dose adjustment based on genotyping of *CYP2C9* could be beneficial to reducing toxicity (Iwamura et al., 2011; Gemmati and Tisato, 2020; Sriram and Insel, 2020; Badary, 2021). Therapeutic response of losartan may also be affected by the *C3435T* SNP of *ABCB1* since a recent study found a significantly increased absorption of losartan in the early phase in patients who carried this variant (Shin et al., 2020).

### 9.7 Janus Kinase Inhibitors

Ruxolitinib is metabolized *via* CYP3A4 and CYP2C9, while baricitinib is metabolized partially by CYP3A4 (Umehara et al., 2019; Takahashi et al., 2020; Veeravalli et al., 2020). Both *CYP3A4* and *CYP2C9* genes are tabulated as VIPs in the PharmGKB database, and certain genetic polymorphisms of these genes may affect the safety or efficacy of the, respective, drugs. Also, the PK of baricitinib may be affected by OAT3 transporter encoded by the *SLC22A8* (Takahashi et al., 2020).

### 9.8 Antibiotics

The PK properties of azithromycin may have interindividual variability due to the variation P-gp expression encoded by the *ABCB1* gene. A single dose of azithromycin had ∼2-fold lower peak concentrations for those who carried rs1045642 SNP of *ABCB1* (TT vs. CC: 468.0 vs. 911.2 ng/ml, *p* = 0.013), as found in 20 healthy volunteers (He et al., 2009). It is important to note that genetic variants of *ABCB1* causing increased concentration of azithromycin may be of particular concern when concomitantly used with HCQ/CQ since the additive effects on QT prolongation may exert fatal arrhythmias (Scherrmann, 2020; Takahashi et al., 2020).

### 9.9 Corticosteroids

Efficacy and toxicities of corticosteroids have been linked to many genetic variants as assessed in various disease conditions. Genes of receptor binding (e.g., *CRHR1* and *NR3C1*), folding proteins (e.g., *ST13*, *STIP1*, and *FKBP5*), metabolic enzymes (e.g., *CYP3A4*, *CYP3A5*, *CYP3A7*, and *GSTT1*), and efflux transporters (e.g., MDR1 and *ABCB1*) may have various genetic polymorphisms accounting for modulating the safety or efficacy of corticosteroids (Song et al., 2017). Pharmacogenetic studies assessing either safety or effectiveness of corticosteroids in either ARDS or COVID-19 were not found in the literature, and it is suggested that the impacts of genetic variants of the genes of interest should focus in future studies in patients with COVID-19 (Takahashi et al., 2020; Vohra et al., 2021).

### 9.10 Antiplatelets

The effects of *CYP2C19* genetic variants on widely used antiplatelets, for example, clopidogrel in either CAD or stroke patients has been well-established including in Thai patients. The findings of these studies suggest that due to the high risk of major adverse cardiovascular events (MACE) such as death, recurrent MI, stroke, and stent thrombosis for patients carrying *CYP2C19* loss-of-function (LoF) alleles, alternative antiplatelets such as prasugrel or ticagrelor not affected by the *CYP2C19* genetic variants should be prescribed in order to reduce the risk of MACE (Sukasem et al., 2013; Biswas et al., 2020a; Biswas et al., 2021b; Biswas et al., 2022; Biswas and Kali, 2021b; Jafrin et al., 2021). The CPIC dosing guidelines already provided clinical recommendations for clopidogrel in acute coronary syndrome (ACS) patients with CYP2C19*2, *3, *17 variants (Scott et al., 2013). In addition to *CYP2C19* genetic variability, magnitude of P-gp expression regulated by the *ABCB1* genetic variants especially *C3435T* SNP of *ABCB1* may also increase the risk of MACE as established in a recent meta-analysis (Biswas et al., 2020b). The episode of stroke or CAD especially MI is considerably high in severe COVID-19 patients (Bikdeli et al., 2020), and it is generally assumed that antiplatelets, for example, clopidogrel is used in these patients as a supportive care; therefore, it is suggested that pharmacogenomic considerations of antiplatelets are highly desirable to optimize the safety or efficacy of these life-saving drugs in severe COVID-19 patients.

### 9.11 Anticoagulants

Anticoagulants, for example, warfarin, have wide interindividual response variability due to the presence of *CYP2C9* and *VKORC1* genetic variants as reviewed elsewhere (Jorgensen et al., 2012; Takeuchi et al., 2020). Both the FDA and CPIC have recommended to consider both the CYP2C9*2, *3 and *VKORC1* (rs9934438) genetic variants for optimizing its safety, that is, bleeding or efficacy in order to achieve precision medicine of warfarin (Dean, 2012; Johnson et al., 2017). Other new oral anticoagulants such as debigatran, rivaroxaban, and apixaban are affected by the *ABCB1* genetic variants and should be considered clinically for optimizing the safety and efficacy (Xie et al., 2018; Kanuri and Kreutz, 2019).

### 9.12 Non-Steroidal Anti-inflammatory Drugs

Non-steroidal anti-inflammatory drugs such as celecoxib, flurbiprofen, ibuprofen, and lornoxicam are predominantly metabolized by CYP2C9 and to a lesser extent by CYP1A2 and CYP3A4. Gastrointestinal (GI) bleeding, myocardial infarction, renal damage, *etc*. are the most common serious adverse effects of NSAIDs; however, many NSAIDs are considered safe and are frequently used as the over-the-counter medicine (Theken et al., 2020). A recent meta-analysis showed that individuals with *CYP2C9* poor metabolizers were associated with significantly increased risk of NSAID-related gastrointestinal bleeding (OR: 1.90, *p* = 0.003) and indicated that CYP2C9*2 was a poor risk predictor, while CYP2C9*3 was a highly significant predictor of GI bleeding (Macías et al., 2020). The CPIC guidelines provided clinical recommendations based on the *CYP2C9* genotype and suggested to consider CYP2C9*2 and CYP2C9*3 variants for patients taking celecoxib, flurbiprofen, ibuprofen, and lornoxicam for optimizing the safety (Theken et al., 2020). Summary of the pharmacogenomics associations of some of the COVID-19 therapeutics with the safety or efficacy is illustrated in Table 3.

**TABLE 3:** Summary of the pharmacogenomic studies affecting the safety or efficacy of COVID-19 therapeutics in other clinical conditions.

COVID-19 therapeutics	Potential genes of interest affecting PK/PD properties	Already assessed gene/genetic variants	Effects on safety or efficacy	Disease condition	Reference
Atazanavir	*UGT1A1* and *ABCB1*	*UGT1A1* and *ABCB1*	Hyperbilirubinemia and dyslipidemia	HIV	(Gammal et al., 2016; Badary, 2021)
	*APOA5*, *APOC3*, *ABCA1*, and *APOE*	*APOA5*, *APOC3*, *ABCA1*, and *APOE*			
Efavirenz	*CYP2B6* and *ABCB1*	*CYP2B6*	Depression and suicidal tendencies	HIV	(Sukasem et al., 2012; Desta et al., 2019)
Oseltamivir	*ABCB1*, *CES1*, *NEU2*, and *SLC15A1*	*CES1* and *ABCB1*	AUC and toxicity	ARDS	(Tarkiainen et al., 2012; Bermúdez de León et al., 2020)
Ivermectin	*ABCB1*, *SLCO1A2*, and *SLCO2B1*	*ABCB1*	Neurologic	Patients with parasite infection	(Baudou et al., 2020; Fricke-Galindo and Falfán-Valencia, 2021)
			Toxicity		
Tocilizumab	FCGR3A, IL6R, CD69, and GALNT18	FCGR3A	Higher response	RA	Takahashi et al. (2020)
Nevirapine	*HLA*, *CYP2B6*, and *ABCB1*	*HLA*, *CYP2B6*, and *ABCB1*	SJS/TEN and hepatotoxicity	HIV	(Martin et al., 2005; Vitezica et al., 2008)
Interferon (INF) β-1b	*IRF6*	*IRF6*	Liver injury	multiple sclerosis	Kowalec et al. (2018)
Azithromycin	*ABCB1*	*ABCB1*	∼2-fold lower peak conc	Healthy volunteers	He et al. (2009)
Clopidogrel	*CYP2C19*	*CYP2C19* and *ABCB1*	MACE	CAD	(Sukasem et al., 2013; Biswas et al., 2020a; Biswas and Kali, 2021b)
	*ABCB1*				
Warfarin	*CYP2C9* and *VKORC1*	*CYP2C9* and *VKORC1*	Efficacy and toxicity	Thrombotic patients	Johnson et al. (2017)
Apixaban, dabigatran, and rivaroxaban	*ABCB1*	*ABCB1*	Efficacy and toxicity	Thrombotic patients	(Xie et al., 2018; Kanuri and Kreutz, 2019)
Losartan	*CYP2C9*	*CYP2C9* and *ABCB1*	Toxicity	Hypertension	(Iwamura et al., 2011; Göktaş et al., 2016)
	*ABCB1*		Efficacy		
NSAIDs	*CYP2C9*	*CYP2C9*	Toxicity	Patients with pain	Theken et al. (2020)

Here, NSAIDs, non-steroidal anti-inflammatory drugs; CAD, coronary artery disease; RA, rheumatoid arthritis; MACE, major adverse cardiovascular events; ARDS, acute respiratory distress syndrome.

## 10 *In Silico* Prediction of Drug Effects in Treatments for COVID-19

To combat COVID-19, computational aided-drug design and screening have been rapidly applied to identify FDA-approved drugs and newly potent compounds from available databases. Using *in silico* approaches, extensive research works have been carried out to acquire an understanding of mechanisms of action and SARS-CoV-2’s activities. However, there are still many foundations to be established for developing novel therapeutics agents for the treatment of COVID-19 (Amin and Jha, 2020). Herein, the current situation in the discovery of anti-SARS-CoV-2 agents at four important targets (Table 4) from *in silico* studies is described and summarized as follows.

**TABLE 4:** Lists of important targets involved in SARS-CoV-2 life cycle mostly used in *in silico* study.

Type		Functions	Important residues	Reference
Host enzymes				
Transmembrane	ACE2	Viral entry	SARS-COV-2 RBD (with corresponding altered residue)	Xiu et al. (2020)
			Cluster 1 (N-terminus); R439, Q498, N501	
			Cluster 2 (central); K417, L455, F456, Y473, Q493	
			Cluster 3 (C-terminus); F486	
Viral enzymes				
Proteases	PLpro (Nsp3)	Catalyzes the viral polyproteins	Catalytic residues; C111, H272, and D286	Amin et al. (2021)
	Mpro (Nsp5)	Catalyzes the viral polyproteins	Catalytic residues; H41 and C145	Amin et al. (2021)
RNA-dependent RNA polymerase	RdRp (Nsp12)	Viral RNA synthesis	Catalytic residues; S759, D760, and D761 (motif C)	Ghazwani et al. (2021)

### 10.1 Spike Protein

**FIGURE 5:**
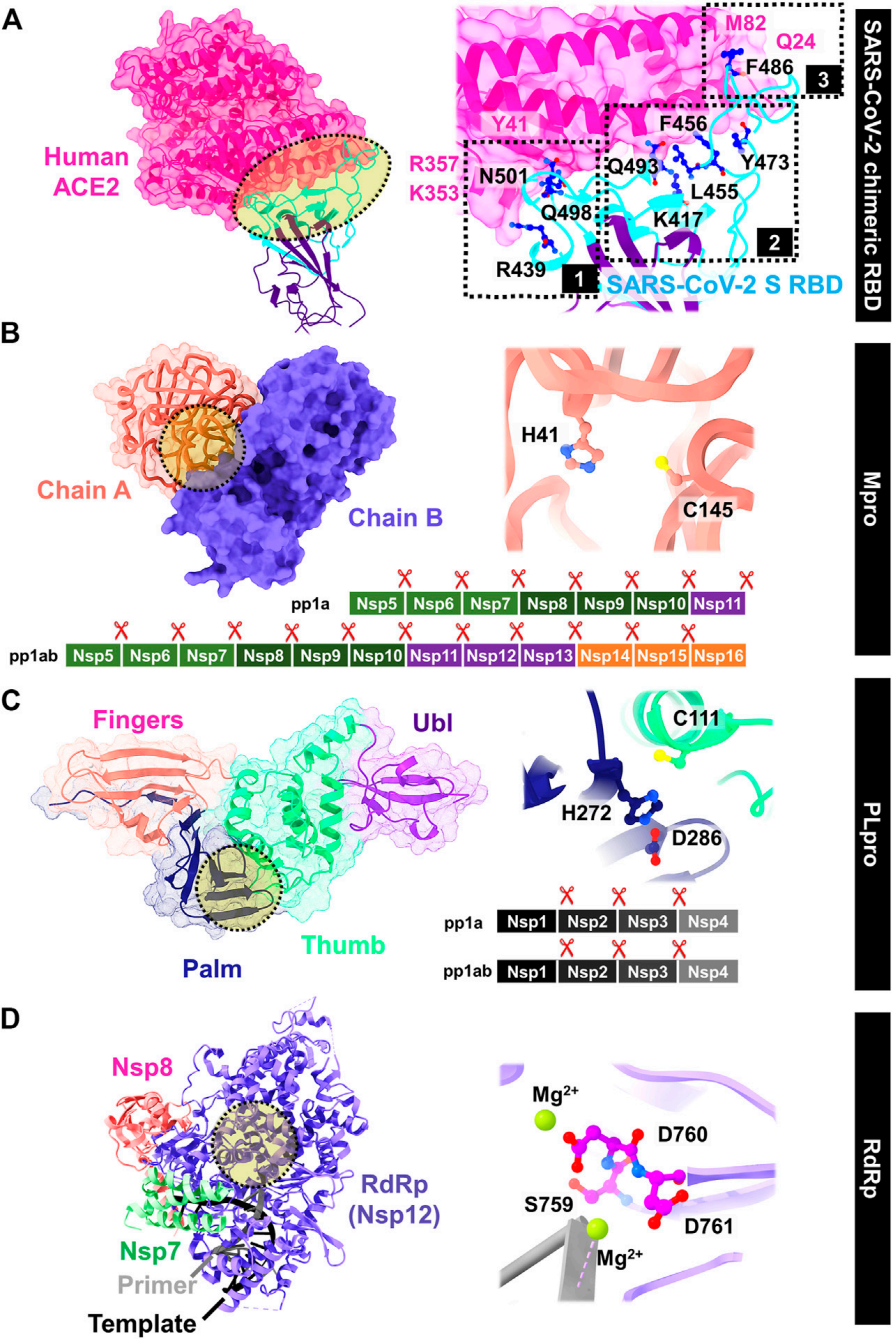
Viral targets for drug development against SARS-CoV-2: **(A)** S RBD/ACE2 binding, **(B)** Mpro, **(C)** PLpro, and **(D)** RdRp, whereas the important residues are also labeled.

SARS-CoV-2 enters into host cells by transmembrane spike (S) glycoprotein that forms homotrimers protruding from the viral surface. The S glycoprotein consists of two subunits responsible for either host cell receptor binding (S1 subunit including the receptor-binding domain, RBD) or the virus fusion (S2 subunit) (Yang et al., 2020). The ACE2 receptor on the host cell is required for viral entering; however, following entry processes vary depending on the cell type (Xiu et al., 2020). The interface can be divided into three parts by mainly polar and is close to the SARS-CoV-2 S/ACE2 complex (Li et al., 2005; Song et al., 2018). In Figure 5A, the extended loop of RBD contacts with ACE2 mainly at the arch-like helix α1 of the proteolytic domain *via* N-terminal, central, and C-terminal (clusters 1–3), and partially at the helix α2 and *β* loops 3–4 (Xiu et al., 2020). The protein–protein binding is likely found at both terminals: 1) formed hydrogen bonds at the α1 N terminus (cluster 1) between the RBD residues Q498, T500, and N501, and the ACE2 residues Y41, Q42, K353, and R357; and 2) van der Waals interactions of Q474 (RBD)--Q24 (ACE2) and F486 (RBD)--M82 (ACE2) at another end (cluster 3). However, only the residue Y453 from the middle cluster 2 contacts with the ACE2 proteolytic domain at residue H34. The SSAA09E2 from the Maybridge HitFinder small-molecule library can inhibit the S-RBD/ACE2 binding (Adedeji et al., 2013), while the chloroquine (Vincent et al., 2005; Wang et al., 2020a) and hydroxychloroquine (Rainsford et al., 2015) used to treat several human diseases including COVID-19 were found to interfere the ACE2.

From molecular docking study on ∼4,000 known drugs from the DrugCentral database (Br et al., 2020) and ∼7,000 antiviral agents from the Asinex database (Farouk et al., 2021) on the S/ACE2 interface followed by molecular dynamic (MD) simulation of screened compounds, the glycyrrhizic acid and the compound 6,612 with the highest binding affinity from the two following databases, respectively, were suggested for further *in vitro* and/or *in vivo* tests. The molecular docking, and physicochemical, pharmacokinetic, and MD studies indicated the solanine, acetoside, and rutin from plant-based natural compounds as the S and Mpro dual inhibitors (Teli et al., 2020; Deetanya et al., 2021). Moreover, several natural herbal compounds such as luteolin, andrographolide, zhebeirine, 3-dehydroverticine, ophiopogonin D, glycyrrhizin, saikosaponin C, crocin-1, and militarine formed strong hydrogen bonds at RBD could prevent the viral binding to ACE2 receptor (Stalin et al., 2021). In addition, the peptide antibiotics (polymyxin B, colistin, and daptomycin), pressure regulators (terlipressin and lypressin), hormone peptides (alarelin and leuprorelin), and immunostimulants (thymopentin) able to hamper the RBD/ACE2 interaction were identified by an *in silico* study. Aurintricarboxylic acid and heparin sodium with binding inhibition of 80 and 63% interacted with RBD at clusters 1 and 2, respectively (David et al., 2021). Computational results could help to demonstrate how geraniin can block the viral entry to human cells by preferentially binding at SARS-CoV-2 S RBD in agreement with the biolayer interferometry-based analysis (Kim et al., 2021).

### 10.2 Proteases

After virion entry into host cells, two polyproteins (pp1a and pp1ab) are translated, which are then divided by two viral proteases: main protease (Mpro) and papain-like protease (PLpro) (Freitas et al., 2020; Goyal and Goyal, 2020). The Mpro, also known as 3C-like protease (3CLpro), has received great attention because of its important involvement in enzymatic activity and post-translational processing of replicase polyproteins. This enzyme has high structural and sequence similarity with SARS-CoV Mpro (Peele et al., 2020; Das et al., 2021b). It contains two catalytic dyad residues C145 and H41 in the active site (Figure 5B) (Zhang et al., 2020c), whereas the residues H41, M49, G143, S144, H163, H164, M165, E166, L167, D187, R188, Q189, T190, A191, and Q192 are involved in substrate binding. The hydrophobic side chains are mainly present at the S2 and S4 sites (Amin et al., 2021). Figure 5C shows the structure of papain-like protease (PLpro) containing the catalytic triad residues C111, H272, and D286 in the active site. The C111 residue engages in Michael addition to the warhead of inhibitors with a formation of a covalent thioether linkage, while the residues Y268, M208, P247, P248, T301, P248, Y264, N267, Q269, L162, C270, G271, and Y273 are involved in substrate binding (Amin et al., 2021).

#### 10.2.1 Main Protease

The *in silico* explorations of potential inhibitors against SARS-CoV-2 Mpro are summarized in Table 5. The first crystal structure of the Mpro with covalent inhibitor N3 was reported in January 2020 (Jin et al., 2020), and the co-crystal data available from many other research groups have provided the basis for fast target-based lead drug development against SARS-CoV-2 Mpro. They were utilized to create a pharmacophore model and perform docking research to identify anti-SARS-CoV-2 inhibitors such as lopinavir, remdesivir, ritonavir, saquinavir, and raltegravir (Daoud et al., 2021). Ritonavir was well occupied in the Mpro active site and interacted with the oxyanion hole residues N142 and G143 (Nutho et al., 2020). Jin et al. (2020) screened >10,000 approved drugs, candidates in clinical trials, and pharmacologically active compounds using combined structure-based virtual and high-throughput screening. The two FDA-approved drugs (disulfiram and carmofur) and four clinical trial compounds (ebselen, tideglusib, shikonin, and PX-12) showed potent SARS-CoV-2 Mpro inhibition with the IC_50_ range of 0.67–21.4 μM. Using the active site conformations of SARS-CoV-2 Mpro through protease pharmacophore clustering, the resulting anti-HCV drugs boceprevir and telaprevir and the anti-HIV drug nelfinavir from a set of 2,122 drugs exhibited significant Mpro inhibition and antiviral efficacy in the micromolar range (Pathak et al., 2021). From the superDRUG2 database, binifibrate and bamifylline identified by e-pharmacophore modeling using the Mpro structure co-crystalized with imidazole–carboxamide inhibitor can bind tightly at the active site and form hydrogen bonds with G143 and E166 throughout MD simulation (Arun et al., 2021). For the mechanism of action for the drug candidates against SARS-CoV-2 Mpro currently studied in clinical trials, the dynamic behavior of PF-07321332 and PF-00835231 showed hydrogen bond formations with C145, E166, and Q189 residues, while additional hydrogen bonds with G143 and H164 were observed in PF-00835231 binding (Ahmad et al., 2021; Baig et al., 2021).

**TABLE 5:** Inhibitors against SARS-CoV-2 Mpro derived from *in silico* screening.

Type of inhibitor	Name	Computational method for study/screening	Binding affinity prediction (kcal/mol)	Method evaluation	IC_50_ (μM)	Reference
FDA-HIV-drug approved	Lopinavir	MD simulations (AMBER)	–10.89	Cell-based assay (HEK-293 T cell infected SARS-CoV-2)	Indeterminable	(Mahdi et al., 2020; Nutho et al., 2020)
	Ritonavir		–14.93		13.7	
FDA-approved drug	Carmofur	Molecular docking (Glide)	**—**	Enzyme-based assay	1.82	Jin et al. (2020)
Clinical trial compounds	Disulfiram				9.35	
	Cinanserin				124.93	
	Tideglusib				1.55	
	Ebselen				0.67	
	Shikonin				15.75	
	PX-12				21.39	
SuperDRUG2 database	Binifibrate	E-pharmacophore model-based virtual screening, MD simulations (Desmond module)	–67.78	**—**	**—**	Arun et al. (2021)
	Bamifylline		–65.24			
Pfizer company	PF-07321332	MD simulations (Gromacs)	−102.00	**—**	**—**	(Ahmad et al., 2021; Baig et al., 2021)
	PF-00835231		**—**			
DrugBank	DB07800	Pharmacophore- and e-pharmacophore, MD simulations (Gromacs)	−54.04	**—**	**—**	T et al. (2021)
	DB03744		−38.83			
	DB03744		−40.25			
	DB02986		−45.01			
	DB03208	Molecular docking (Glide)	−15.07	**—**	**—**	Debnath et al. (2021)
	DB03949		−10.91			
	DB08001		−10.15			
	DB08526		−9.30			
	DB02558		−8.94			
	DB12332		−8.78			
	DB02651		−8.70			
FDA-approved drug	Boceprevir	Combined protease pharmacophore clustering and molecular docking (iGEMDOCK)	−25.7	Enzyme-based assay	1.63	Pathak et al. (2021)
				Antiviral activity (Vero cell infected SARS-CoV-2)	49.89 (EC_50_)	
				Cytotoxicity (Vero E6 cell)	159.6 (CC_50_)	
	Telaprevir		−5.5	Enzyme-based assay	11.47	
				Cytotoxicity (Vero E6 cell)	35.8	
Drugs and Probes database	Myricetin	Molecular docking (Glide)	**—**	Enzyme-based assay (FRET assay)	0.22	Maria et al. (2021)
	Thioguanosine				6.3	
	MG-132				7.4	
	Bronopol				0.4	
	Sennoside A				1.59	
	ML311				0.15	
	PR-619				0.41	
	Felbinac ethyl				0.20	
	Z-DEVD-FMK				0.01	
	Oltipraz				0.21	
Natural drugs from ZINC database	Daidzin	Pharmacophore mapping and MolDock	−115.11	**—**	**—**	Saeed et al. (2021)
Peptidomimetic	N3	MD simulations (AMBER)	−9.92	Cell-based assay (qRT–PCR in Vero cell infected SARS-CoV-2)	16.77	(Jin et al., 2020; Amin et al., 2021; Somboon et al., 2021)
	11a		−9.68	Enzyme-based assay	0.053	
	13b		−10.35	Enzyme-based assay	0.67	
	14b		−9.64	**—**	**—**	
Anthocyanin-derived compounds from the PubChem database	ID44256891	Structure-based pharmacophore modeling PHASE module and molecular docking (Glide)	−12.37	**—**	**—**	Fakhar et al. (2021)
	ID 44256921		−11.59			
	ID102452140		−10.94			
	ID131751762		−10.30			
	ID131831710		−13.59			
	ID139031086		−9.58			
N-phenyl-2-(pyrimidin-2-ylthio)acetamide analogs	N-(3,4-dichlorophenyl)-2-(5-(2-phenylthiazol-4-yl)pyrimidin-2-ylthio)acetamide	Virtual screening and 3D-QSAR	**—**	Edans-Dabcyl FRET assay	3.0	Tsai et al. (2006)
MolPort compound library	M-8524	Combined virtual screening, MD simulations, and machine learning	**—**	Enzyme-based assay (FRET assay)	31.0	Glaab et al. (2021)

Some natural products with promising pharmacodynamic and pharmacokinetic characteristics, for example, higenamine hydrochloride, phloretin, daidzin, and naringenin chalcone, were screened from the ZINC database using the receptor-based pharmacophore modeling and molecular docking (Saeed et al., 2021). The receptor-, ligand-, and machine learning-based screening methods elucidated the small-molecule inhibitors of Mpro with IC_50_ in the micromolar range: rottlerin (37 μM), amentoflavone (143 μM), baicalein (208 μM), and synthetic compounds (e.g., CID 46897844, 31 μM) (Glaab et al., 2021). The crucial residues frequently participating within these potent compounds are E166, T190, Q189, and Q192, while the catalytic residues H41 and C145 are important for amentoflavone and baicalein, respectively. The inhibitors such as N3 and myricetin which covalently bound to C145 could terminate the SARS-CoV-2 Mpro functions (Jin et al., 2020; Glaab et al., 2021). The MD study on Mpro in complex with the four reported peptidomimetic inhibitors N3, 11a, 13b, and 14b indicated that the ligand–protein complexation is mainly driven by vdW and hydrogen bond interactions (Somboon et al., 2021). The polar moieties (e.g., benzamide) and the bulky N-terminal protecting groups (e.g., thiazole) should be introduced to P1’ and P4 sites of 13b structure to increase hydrogen bonds and hydrophobic interactions, respectively.

#### 10.2.2 Papain-Like Protease

The two irreversible inhibitors VIR250 and VIR251 with a significant degree of SARS-CoV-2 PLpro selectivity over other proteases were discovered (Rut et al., 2020), and their crystal structures were widely used for virtual screening. The PLpro inhibitors derived from *in silico* screening are illustrated in Table 6. The antidiabetic drug phenformin, anti-HIV drug ritonavir, and natural compound quercetin resulted from ∼1,700 clinical FDA-approved drugs showed favorable pharmacokinetics and strong binding interactions with SARS-CoV-2 PLpro (Kandeel et al., 2021). In addition to quercetin, PLpro has also been shown to bind with several compounds from the 26 Chinese herbal medicines such as cryptotanshinone and tanshinone IIa (Zhang et al., 2020b). Some anti-HCV drugs, namely, simeprevir, grazoprevir, and vaniprevir, with PLpro inhibition synergize with remdesivir to reduce SARs-CoV-2 virus replication in Vero and/or human cells (Bafna et al., 2021).

**TABLE 6:** Inhibitors against SARS-CoV-2 PLpro derived from *in silico* screening.

Type of inhibitor	Name	Computational method for study/screening	Binding affinity prediction (kcal/mol)	Method evaluation	IC_50_ (μM)	Reference
FDA-approved drug	Phenformin	MD simulations (AMBER)	−56.5	**—**	**—**	Kandeel et al. (2021)
	Quercetin		−40.9			
	Ritonavir		−37.6			
	Montelukast		−36.4			
	Fostamatinib 1		−33.5			
	Candesartan		−28.9			
Chinese herbal medicine	Cryptotanshinone	Molecular docking (AutoDock Vina)	−5.25	**—**	**—**	Zhang et al. (2020b)
	Tanshinone IIa		−5.02			
PubChem and ZINC databases	Deoxycylindrospermopsin	MD simulations (Gromacs)	−41.39	**—**	**—**	Naidoo et al. (2021)
ENAMINE REAL database	rac3j	Pharmacophore model-based virtual screening	**—**	Fluorescence polarization-based PLpro activity	1.40	(Freitas et al., 2020; Klemm et al., 2020; Stasiulewicz et al., 2021)
	rac3k				1.15	
	rac5c				0.81	
	6577871			Enzyme-based assay	100.7	
	7724772				23.5	
	Compound 6				5.0	
Antiviral secondary metabolites from fungi	Norquinadoline A	Molecular docking (UCSF	−10.9			Quimque et al. (2021)
	Asperterrestide A		−8.9			
	Rubrolide S		−8.7			
	Isoaspulvinone		−7.7			
	Deoxynortryptoquivaline		−9.6			
	Arisugacin A		−10.0			
	Isochaetochromin D1		−9.9			
	Penicillixanthone A		−9.5			

The naphthalene-based derivatives with previously reported SARS-CoV-1 PLpro inhibitory activity could be beneficial for SARS-CoV-2 treatment (IC_50_ values of 2.4 and 5 μM for GRL-0617 and compound 6, respectively) due to almost identical residues in the PLpro BL2 loop of the two viruses (Amin et al., 2021). The selective compounds from the ENAMINE REAL database using pharmacophore modeling which have IC_50_ values of 159–505 nM bind to the target protein in a similar manner to the non-covalent SARS-CoV-2 PLpro inhibitor, GRL-0617 (Stasiulewicz et al., 2021). The identified deubiquitinase inhibitors against PLpro, TCID and DUB-IN-3 with IC_50_ of 6.42 and 12.5 μM, formed hydrogen bonding with the PLpro residues Y264 and R166, respectively (Liu et al., 2021). The *in silico* molecular interaction-based method was used to elucidate the cyanobacterial metabolites against SARS-CoV-2 PLpro. The deoxycylindrospermopsin binding with the important residues T26, C44, F140, S144, C145, H163, and E166 was identified as the most promising inhibitory candidate (Naidoo et al., 2021). By molecular docking and MD study of 97 antiviral secondary metabolites from fungi, norquinadoline A was found to be the most effective inhibitor of SARS-CoV-2 PLpro with high gastrointestinal absorption, low blood–brain barrier penetrability, and high drug-likeness (Quimque et al., 2021).

### 10.4 RNA-Dependent RNA Polymerase

RNA-dependent RNA polymerase or RdRp (Nsp12) catalyzes viral RNA synthesis, and as a result, it plays a key role in viral replication and multiplication, alongside cofactors Nsp7 and Nsp8 proteins. Among seven key motifs in RdRp catalytic domain, motifs A–F are highly conserved across all viral RdRps, but motif G is a unique structural characteristic of primer-dependent RdRps in some positive-sense RNA viruses binding with the primer strand at the beginning of RNA synthesis. In Figure 5D, the catalytic residues S759, D760, and D761 with Mg^2+^ as a catalyst cofactor are located in motif C (Naydenova et al., 2021), while the other crucial residues are D618, C622, and D623 in the active site, S682, T687, A688, and N691 in motif B, and K545, R553, and R555 in motif F (Ghazwani et al., 2021).

Favipiravir is the first antiviral drug authorized for the treatment of SARS-CoV-2 by China’s National Medical Products Administration. Several drugs, that is, sofosbuvir, ribavirin, galidesivir, and remdesivir, are being tested in clinical trials against SARS-CoV-2 RdRp (Elfiky, 2020). In the reported crystal structures, the drugs favipiravir and remdesivir are accommodated in the ATP binding site of RdRp (Yin et al., 2020; Naydenova et al., 2021). *In silico* drug design and discovery of RdRp inhibitors are given in Table 7. In addition to remdesivir and ribavirin, the molecular docking study of 1,749 antiviral drugs suggested that paritaprevir, glecaprevir, and velpatasvir also showed interesting interactions with RdRp (Singh et al., 2021). The top 50 compounds retrieved from structure-based virtual screening of 15,220 compounds from DrugBank and TargetMol Bioactive compounds Library against SARS-CoV-2 RdRp were evaluated by bio-layer interferometry (BLI) binding followed by cell-based polymerase activity assays (Li et al., 2021). Corilagin showed the highest inhibition SARS-CoV-2 RdRp (K_D_ of 0.54 220 μM) and inhibited viral replication in Vero cells (EC_50_ of 0.13 μM), by binding at RdRp’s palm domain and thus preventing the conformational changes necessary for nucleotide incorporation. Its binding pocket comprised the conserved residues S759, D760, and D761 in motif C, and the surrounding residues G616, D761, K798, W61, W800, D618, S814, E811, S549, C799, and A550. Relative to remdesivir, the 11 obtained compounds from the virtual screening and MD simulations derived from the ZINC database displayed significant interactions with all RdRp active site residues (Ghazwani et al., 2021). Based on pharmacophore modeling of the remdesivir/RdRp complex (two anionic acceptor, one donor, one acceptor, and one dual donor and acceptor features), the epigallocatechin gallate, kuromanin, procyanidin-b-2, and rutin were the top four hits among 5,836 compounds from the ChEMBL database (Kandwal and Fayne, 2020). Pharmacophore modeling, structure-based virtual screening, and MD simulations of RdRp bound with the known RdRp inhibitors were used to screen the potential agents from the six databases; PubChem-134297651, CHEMBL387201, CHEMBL1196124, PubChem-122704503, ZINC257357489, and ZINC5605331, which were highly interacting with RdRp at the ATP binding pocket (Grzybowski et al., 2002).

**TABLE 7:** Inhibitors against SARS-CoV-2 RdRp derived from *in silico* screening.

Type of inhibitor	Name	Computational method for study/screening	Binding affinity prediction (kcal/mol)	Method evaluation	IC_50_ (μM)	Reference
DrugBank and TargetMol Bioactive compounds Library	Verbascoside	Molecular docking (AutoDockTools), MD simulations (AMBER)	−8.3	Bio-layer interferometry (BLI) binding assay	0.84 (K_D_)	Li et al. (2021)
	Oleanonic acid		−9.6		171.00	
	Forsythoside A		−8.7		1.61	
	MK-3903		−8.9		220.00	
	T-5224		−8.6		38.70	
	Corilagin		−8.9		0.54	
				Cell-based assay (Vero cell infected SARS-CoV-2)	0.13 (EC_50_)	
				Cell-based assay (Huh-7 cell infected HCoV-OC43)	2.49 (EC_50_)	
	Remdesivir		—	Cell-based assay (Vero cell infected SARS-CoV-2)	0.06 (EC_50_)	
				Cell-based assay (Huh-7 cell infected HCoV-OC43)	4.96 (EC_50_)	
ZINC Drug Database and DrugBank	Favipiravir	Virtual screening and molecular docking (Glide; induced-fit docking)	−4.8	Cell-based assay (Vero cell infected SARS-CoV-2)	22.50 (EC_50_) >100 μM (CC_50_)	(Wang et al., 2020a; Poustforoosh et al., 2021)
	Ribavirin		−6.9		109.5 (EC_50_) >400 μM (CC_50_)	
	Galidesivir		−7.1	—	—	
	Tenofovir		−5.7			
	Valganciclovir		−5.0			
	Ceftibuten		−5.1			
	Fenoterol		−7.5			
	Silybin		−6.9			
	Idarubicin		−7.9			
Approved small-molecule drugs	Dexamethasone metasulfobenzoate	Molecular docking (AutoDock VINA)	−8.7	—	—	(Jeon et al., 2020; Khater et al., 2021)
	Conivaptan		−8.6	Vero cell infected SARS-CoV-2	10	
	Dutasteride		−8.6	—	—	
	Hesperidin		−8.6			
	Lumacaftor		−8.6			
	Glycyrrhizic acid		−8.6			
	Dexamethasone metasulfobenzoate		−8.6			
	Ergotamine		−8.5	Predicted IC_50_	190	
	Eltrombopag		−8.5	Vero cell infected SARS-CoV-2	8.0	
					>50 μM (CC_50_)	
	Astemizole		−8.4	—	—	
	Chlorhexidine		−8.4			
	Gliquidone		−8.4			
ChEMBL, Chem Div, Molport, the NCI open Chemical repository, PubChem, ZINC purchasable databases	PubChem-134297651	Pharmacophore-based virtual screening (HipHop algorithm) and molecular docking (Gold)	72.61 (Gold score)	—	—	Fayyazi et al. (2021)
	CHEMBL387201		64.85 (Gold score)			
	CHEMBL1196124		64.34 (Gold score)			
	PubChem-122704503		61.25 (Gold score)			
	ZINC257357489		47.44 (Gold score)			
	ZINC5605331		40.29 (Gold score)			
	MAW-22	Fragment-based drug design, docking, MD simulations (Gromacs)	−390.24			El Hassab et al. (2020)
ChEMBL database	Epigallocatechin gallate	Pharmacophore-based virtual screening (MOE Module)	—	—	—	Kandwal and Fayne, (2020)
	Kuromanin					
	Procyanidin-b-2					
	Rutin					

## 11 Pharmacogenomics for COVID-19 Vaccine

At least 13 different vaccines have been administered until now to combat the biggest infectious challenges of the 21st century. Although majority of these vaccines are well-tolerated, they may not be responsive similarly to everyone and may also account for some vaccine-related side/toxic effects. The application of existing pharmacogenetic/pharmacogenomic science to vaccines is termed as “vaccinomics” (Hoffman et al., 1998; Poland et al., 2007, 2021; Omersel and Karas Kuželički, 2020; Soiza et al., 2021). Since the pharmacogenomic association with the safety or efficacy of many clinical important medications, for example, antiepileptics, antiplatelets, cardiovascular drugs, antidepressants, and anticancers, have been well-established and many of these considerations are now implemented in routine clinical practice in some parts of the world, we are expecting that similar approaches in terms of COVID-19 vaccines will start to appear very soon.

Some genetic insights for vaccines applied in other infectious diseases have already been recognized, for example, specific genetic polymorphisms of the *TLR3* gene were associated with significantly reduced immune responses to the measles vaccine as reviewed elsewhere (Poland et al., 2018). Vaccinomics provide a promising newly evolving research area through which the safety or efficacy of vaccines may be optimized. A wide range of genotype/phenotype association data are currently being integrated into this newly emerging research field for many live viral vaccines and expecting that similar attributes will begin for COVID-19 vaccines as well. The application of vaccinomics in COVID-19 may allow us to explain the interindividual immune responses’ variability and adverse events, and may also accelerate the development of personalized vaccine (Poland et al., 2021).

Immunogenicity data of COVID-19 vaccination come from the assessment of specific T-cell responses and specific antibody responses (Zhu et al., 2020). Both activation of cytotoxic T cell and antibody production need antigen presentation *via* HLA class I and II, respectively. We hypothesize that variation of the *HLA* genotype might affect the immunogenicity of COVID-19 vaccines. Further investigation in association between *HLA* polymorphism and COVID-19 vaccine immunogenicity is interesting and might help to predict individual COVID-19 vaccine effectiveness.

Within a year, several vaccines have been developed and millions of doses were delivered. The ChAdOx1 nCoV-19 vaccine (AstraZeneca) has recently been reported as an increasing risk of venous thrombosis and thrombocytopenia, called vaccine-induced immune thrombotic thrombocytopenia (VITT), 7–10 days after receiving the first dose (Schultz et al., 2021). Recently, some studies showed that pathogenic antibodies to platelet factor 4 (PF4), which have a major role to develop VITT, can occur after the administration of the ChAdOx1 nCoV-19 vaccine (Scully et al., 2021). This pathogenic PF4-dependent syndrome is unrelated to the use of heparin therapy, called heparin-induced thrombocytopenia (HIT). The higher PF4 level has an association with HLA-DRB1*03:01-DQB1*02:01 haplotype (Zhang et al., 2019). Genetic variants of *HLA-DRB1* and *HLA-DQB1* were found ∼25% in Thai population (Puangpetch et al., 2014; Satapornpong et al., 2020), indicating that considerable proprotion of Thai population might be at risk of developing VITT associated with these genetic polymorphisms. Clinical validation of the screening of HLA-DRB1*03:01 and HLA-DQB1*02:01 to predict the occurrence of VITT should be investigated, and it might help people to avoid this life-threatening condition. Even though platelet level is very low, VITT should not be treated by platelet transfusion. Ideally, such transfusion should be avoid because it would provide a substrate for further antibody-mediated platelet activation and coagulopathy. Therefore, rapid recognition of VITT is very important.

## 12 Precision Medicine for COVID-19 Treatment: Key Clinical Considerations

### 12.1 Drug–Drug Interactions

It is also alarming that many patients are developing serious complications, for example, life-threatening adverse drug reactions. Since the mortality of COVID-19 patients is significantly higher in older patients and also for those having multiple comorbidities (Biswas et al., 2021a), it is likely to expect DDIs and consequently ADRs generated from the risk of polypharmacy, as evidenced in recent observational studies (Ramírez et al., 2020; Falcão et al., 2021). For example, at least 62 life-threatening potential DDIs of LPV/RTV that should be considered in COVID-19 patients if taking these drugs have been identified (Biswas, 2020). A summary of very important clinically significant DDIs of COVID-19 therapies as evidenced or suggested elsewhere is shown in Table 8.

**TABLE 8:** Potential clinically significant DDIs of COVID-19 therapeutics.

COVID-19 therapeutics	Interacting drug/herb/fruits	Effects of DDI/DHI	Reference
Ribavirin	Warfarin	Decrease anticoagulant effects of warfarin	Rezaee et al. (2021)
Ribavirin	Azathioprine (AZA)	Increase risk of f myelotoxicity, that is, anemia, and thrombocytopenia of AZA	Rezaee et al. (2021)
Lopinavir/ritonavir	Amiodarone	Cardiac toxicity and QT prolongation	Rezaee et al. (2021)
	Dronedarone		
	Flecainide		
	Ivabradine		
	Propafenone		
	Mexiletine		
Lopinavir/ritonavir	Rivaroxaban	Risk of bleeding	Rezaee et al. (2021)
Lopinavir/ritonavir	Simvastatin	Risk of rhabdomyolysis	Rezaee et al. (2021)
Lopinavir	Alfuzosin	Increase QT interval	Rezaee et al. (2021)
Lopinavir/ritonavir	Rifampicin	Increase risk of hepatocellular toxicity	Rezaee et al. (2021)
Lopinavir/ritonavir	Salmeterol	Increase cardiac complications	Rezaee et al. (2021)
Tocilizumab	Dabigatran and etexilate	Risk of thrombosis	Rezaee et al. (2021)
Tocilizumab	Adalimumab	Risk of serious infection and immunosuppressive effects	Rezaee et al. (2021)
Remdesivir	Rifampicin	Increase the risk of hepatotoxicity	Rezaee et al. (2021)

DDIs are likely important in case of assessing pharmacogenomic effects of any particular drug since sometimes the DDI may alter/exacerbate the clinical effects or change phenotypes (phenoconversion) associated with interactions with the, respective, genes (Klomp et al., 2020). A recent systematic review reported a phenoconversion from a higher metabolizer phenotype into a lower metabolizer phenotype by the concurrent use of CYP inhibiting drugs and also by the extrinsic factors such as cancer, inflammation, and older age. By contrast, phenoconversion from a lower metabolizer phenotype into a higher metabolizer phenotype was reported by the concomitant use of CYP inducer drugs and also by smoking. In addition, alcohol, pregnancy, and vitamin D exposure may also contribute to the phenoconversion process (Klomp et al., 2020).

If any patients are taking COVID-19 therapeutics influenced by the CYP metabolism and taking CYP inhibitors, also carrying *CYP* genetic variants, then the net clinical effects may be further exacerbated profoundly. There is evidence for such phenomenon as reported in a recent meta-analysis showing that for patients carrying *CYP2C19* LoF alleles and taking clopidogrel and proton pump inhibitors, the risk of MACE was almost over double compared to the patients taking clopidogrel with or without *CYP2C19* LoF alleles (Biswas et al., 2021b). Therefore, DDIs are very important clinical considerations for optimizing safety or effectiveness of COVID-19 medications through assessing pharmacogenomic interventions.

### 12.2 Patients’ Condition and Underlying Diseases

Systemic vascular inflammation and coagulopathy resulting from cytokine storm contribute to multi-organ failure in patients with severe COVID-19. In addition to the genetic biomarker, that is, *HLA* genotype, the non-genetic biomarkers associated with the severity and disease progression of COVID-19 can be divided into 1) hematological biomarkers [lymphocyte count, neutrophil count, and neutrophil–lymphocyte ratio (NLR)], 2) inflammatory biomarkers [C-reactive protein (CRP), erythrocyte sedimentation rate (ESR), and procalcitonin (PCT)], 3) immunological biomarkers (IL-6 and IL-10), 4) biochemical biomarkers [D-dimer, troponin, creatine kinase (CK), and aspartate aminotransferase (AST)], and 5) new laboratory biomarkers (homocysteine and angiotensin II) (Ponti et al., 2020a). Almost all these biomarkers are previously used to monitor the critically ill patients with systemic infection/inflammation or multi-organ failure from several causes.

The levels of cytokine storm are associated with COVID-19 severity and severe progression. Among them, IL-6 and IL-10 could be used as biomarkers for fast diagnosis of patients with a higher risk of disease deterioration (Han et al., 2020). The identification of IL-6 might also potentially benefit from anti-IL-6 immunotherapies with tocilizumab (Zhang et al., 2020a).

The level of D-dimer or fibrin degradation products (FDPs) is a strong evidence of thrombosis and thromboembolism (Halaby et al., 2015). Studies have reported an increase in D-dimer and fibrinogen concentrations in the early stages of COVID-19 disease; a 3 to 4-fold rise in D-dimer levels is linked to poor prognosis (Rostami and Mansouritorghabeh, 2020). Monitoring the level of D-dimer might help in determining the prognosis of patients and making the decision of early aggressive treatment.

Plasma levels of homocysteine are associated with vascular inflammation and damage (Balint et al., 2020). Recent data demonstrated a predictive value of homocysteine for the severity of pneumonia from COVID-19 (Ponti et al., 2020b). The high plasma level of Ang II also has been demonstrated in COVID-19 patients with severe lung injury and high viral load (Gheblawi et al., 2020; Liu et al., 2020). When we get further clinical validation, these two new biomarkers would be useful to predict or determine the severity of pneumonia in COVID-19 patients.

### 12.3 Drug–Herb Interactions

Medicinal plants often serve as a very crucial alternative or adjuvant therapy to synthetic allopathic drugs for combating numerous diseases from the ancient time. Andrographolide isolated form the medicinal plant of *Andrographis paniculata* has been clinically used for the treatment of inflammatory diseases and viral infections for many years. Recent molecular docking suggests that andrographolide is able to form a covalent bond with the active site of SARS-CoV-2 and may suppress the progression of this pandemic virus. This herbal component may therefore serve as an alternative option for the management of the COVID-19 pandemic (Shi et al., 2020b).

However, interaction of andrographolide with other drugs called drug–herb interactions (DHIs) might be of particular interest in assessing its potency against SARS-CoV-2 infection. An *in vitro* study found potent inhibitory effects on the activities of CYP3A4 and CYP2C9 enzymes by andrographolide, suggesting that drug–herb interactions (DHIs) of andrographolide would be of particular concern for drugs primarily metabolized by CYP3A4 and CYP2C9 pathways such as warfarin (Pan et al., 2011). This is consistent with the findings of another study suggesting that andrographolide could cause DHIs in humans through interfering of CYP2C9 or CYP3A4 enzyme activities (Pekthong et al., 2008, 2009). DHI between andrographolide and warfarin has already been established in the mouse model where andrographolide was found to increase the systemic exposure of warfarin probably by the inhibition of the CYP3A4- or CYP2C9-mediated warfarin metabolism (Zhang et al., 2018). Another study found a DHI between andrographolide and tolbutamide where andrographolide enhanced the metabolic rate of tolbutamide by increasing the expression and activity of certain CYP enzymes (Chen et al., 2013). Also, andrographolide could also induce CYP1A1 and CYP1A2 expression as found in the mouse model (Jaruchotikamol et al., 2007), potentially interacting with drugs metabolized by CYP1A1 or CYP1A2. Many other herbs may also interact with COVID-19 therapies as shown in Table 9.

**TABLE 9:** Potential DHIs of COVID-19 therapeutics and herbs.

COVID-19 therapeutics	Interacting herbs/components	Effects of DHIs	Involvement of CYP enzymes	Reference
Lopinavir/ritonavir	Qingfei Paidu decoction (QPD) consisting of 20 herbs	*In vivo* study revealed that QPD extended the half-life of lopinavir by 1.40-fold and raised the AUC by 2.04-fold	Through strong inhibition of CYP3A	Zhang et al. (2021a)
Lopinavir/ritonavir	*Echinacea purpurea*	Although PK of these combination drugs was not affected significantly, it may affect when using CYP3A4 substrate drugs	Induce CYP3A	Penzak et al. (2010)
Lopinavir	*Ginkgo biloba*	May reduce drug exposure	Induce CYP3A	Robertson et al. (2008)
Efavirenz and nevirapine	*Hyptis suaveolens*, *Myrothamnus flabellifolius*, *Launaea taraxacifolia*, and *Boerhavia diffusa*	Risk of drug toxicity	Inhibit CYP2B6	Thomford et al. (2016)
Nevirapine	*Hypericum perforatum*	Increase plasma concentration of nevirapine	Induce CYP2B6	Chen et al. (2012)
Atazanavir	*Astragalus membranaceus*	Weak herb–drug interaction	Weak inhibition of CYP3A2	Cheng et al. (2015)
Darunavir/ritonavir	*Echinacea purpurea*	Decrease darunavir concentration	Induce CYP3A4	Moltó et al. (2011)
Losartan	*Silymarin*	Reduce metabolism	Inhibit CYP2C9	(Han et al., 2009; Rombolà et al., 2020)
Indinavir	*Hypericum perforatum*	Decrease plasma concentration of indinavir by 57%	Induce CYP3A4	Chen et al. (2012)
Lopinavir	HEJG consisting of nine herbs	Significantly increase the plasma level of lopinavir by 2.43-fold	Inhibit CYP3A	Zhang et al. (2021b)
Warfarin	*Andrographis paniculata*	Increase systematic exposure of warfarin	Inhibit CYP2C9, CYP3A4	(Pan et al., 2011; Zhang et al., 2018)
	*Ginkgo biloba*	Increase bleeding risk	Inhibit CYP2C9	(Chen et al., 2012; Asher et al., 2017)
Clopidogrel	St John’s wort	Increase responses of clopidogrel	Induce CYP2C19	Rahimi and Abdollahi, (2012)
	*Ginkgo biloba*	Increase risk of bleeding	Through CYP2C19	(Aruna and Naidu, 2007; Deng et al., 2016; Asher et al., 2017)

Here, AUC, area under the concentration–time curve.

In addition of DHIs, we are also concerned about the pharmacogenetics of herbs termed as “herbogenomics,” potentially affecting the safety or efficacy of herbs. For examples, genetic polymorphisms of *CYP3A4* or *CYP2C9* may modify the clinical effects of andrographolide and should be considered clinically along with DHIs. Although the concept is new, it suggests to considering herbogenomics in future studies for patients taking COVID-19 medications. In addition to andrographolide, many other herbs are using against SARS-CoV-2 infection, as shown in Table 10.

**TABLE 10:** Herbs using against COVID-19 management.

Herb name/scientific name	Active herb components/whole herbs	Potential mechanism of action against SARS-CoV-2	Reference
*Andrographis paniculate*	Andrographolide	Forms a covalent bond with the active site of SARS-CoV-2 and may suppress the progression as found in a recent molecular docking study	Shi et al. (2020b)
Turmeric (*Curcuma longa*)	Curcumin	Inhibits the host entry of SARS-CoV-2 by interfering viral S protein and host ACE2 receptor protein	Das et al. (2021a)
*Eucalyptus globulus*	Citronellol, alpha-terpineol, *o*-cymene, d-limonene, eucalyptol, alpha-pinene, and 3-carene	*In silico* study reported potential inhibitor of SARS-CoV-2 M^pro^	Panikar et al. (2021)
*Corymbia citriodora*			
Garlic (*Allium sativum*)	Allicin and allitridin	Interacts with SARS-CoV-2 M^pro^ protease	(Donma and Donma, 2020; Rouf et al., 2020)
*Houttuynia cordata*	Alkaloids, polyphenols, and flavonoids	Inhibits RdRp	(Das et al., 2021a; Bahadur Gurung et al., 2021)
Ginger (*Zingiber officinale*)	24-Methylcholesta-7-en-3β-on, spinasterone, and spinasterol	Inhibits SARS-CoV-2 3CL protease enzyme	Zubair et al. (2021)
Strawberry (*Fragaria ananassa* Duch.)	With silver nanoparticle (AgNPs)	Demonstrated marked activity against SARS-CoV-2	Al-Sanea et al. (2021)
Ginger (*Zingiber officinal*)	Neohesperidin	*In silico* study demonstrated that neohesperidin potentially binds to both human AAK1 protein and SARS-CoV-2 NSP16 protein	
*Andrographis paniculata*	Diterpene, flavonoids, and aglycone/glycoside	Inhibits SARS-CoV-2 M^pro^ protease	Sukardiman et al. (2020)
Licorice or Glycyrrhizae (GR)	Flavonoids and terpenes/saponins	May modify TNF and IL-17 signaling pathways, and helps to overcome SARS-CoV-2 infection	Ng et al. (2021)
Licorice (*Glycyrrhiza glabra*)	Glycyrrhizin (GR) and glycyrrhetinic acid (GA)	GR can interfere with virus entry by directly interacting with ACE2 and spike	Diomede et al. (2021)
*Siparuna cristata*	Retusin and kumatakenin	*In silico* found inhibitory effect against 3CLpro and PLpro SARS-CoV-2 protease	Leal et al. (2021)
*Reynoutria Rhizomes*	Procyanidins and anthranoids	Strong inhibitor of SARS-CoV-2 M^pro^	Nawrot-Hadzik et al. (2021)
Ashwaganha *Withania somnifera*	Withanoside V, somniferine tinocordiside, vicenin, isorientin 40-O-glucoside 200-O-*p*-hydroxybenzoagte, and ursolic acid	These phytochemicals bind with SARS-CoV-2 M^pro^	Shree et al. (2020)
*Tinospora cordifolia* (giloy)			
*Ocimum sanctum* (tulsi)			
*Allium sativum* (garlic)	Allicin and allitridin	Interacts with the M^pro^ protease	(Donma and Donma, 2020; Rouf et al., 2020)
*Vitex negundo* and *Justicia adhatoda*	Eudesmol and viridiflorene	Target of SARS-CoV-2 (M^pro^, ACE-2, S-protein, and RdRp as reported in the *in silico* study	Gowrishankar et al. (2021)
*Eucalyptus globules*	Ellagic acid and apigenin-7-O-glucuronide	Inhabits SARS-CoV-2 M^pro^	
*Justicia adhatoda*	Anisotine and vasicolinone	Blocks viral replications	
*Theobroma cacao*	Amentoflavone, naringin isorhoifolin N3, rutin, and isorhoifolin	Revealed activity to interfere with M^pro^	Yañez et al. (2021)
		Isorhoifolin and rutin seem to bind more strongly than N3 co-crystallized inhibitor	
Citrus fruits	Hesperidin and hesperetin	Halts the interaction between viral S protein and ACE2 receptor and suppresses the ACE2 and TMPRSS2 expression	Cheng et al. (2021)
Lianhuaqingwen (LH) capsule	Consists of several plants including *Lonicera japonica* and *Forsythia suspense*	*Lonicera japonica* and *Forsythia suspensa* could block the binding of SARS-CoV-2 with ACE2	Hu et al. (2021b)
		LH conferred suppression of the cytopathic effect of SARS-CoV-2 *in vitro* and reduced the viral loads in the cytoplasm and cellular membrane	Runfeng et al. (2020)
*Nigella sativa* (black seed)	Phenolic compounds, flavonoids, phytosterols, alkaloids, glycosides, and volatile oils	It affects binding at the site of N3 in the SARS-CoV-2 M^pro^	(Maideen, 2020; Puttaswamy et al., 2020)
*Eucalyptus globulus*	Apigenin-7-O-glucuronide (AG) and ellagic acid from leaves	*In silico* study reported the inhibitory effects against M^pro^, ACE-2, S-protein, and RdRp. AG inhibits RdRp complex higher than remdesivir	Gowrishankar et al. (2021)
*Justicia adhatoda*	Vasicolinone and Anisotine		
*Vitex negundo*	Eudesmol and viridiflorene		
*Murraya koenigii* (L.) Spreng	Bismahanine	Binds with the spike protein	Puttaswamy et al. (2020)
*Hypericum perforatum L.*	Hypericin	Inhibits SARS-CoV-2 M^pro^	
*Cephalotaxus wilsoniana* Hayata	Taiwanhomoflavone A	Targets RdRp at 9.8 6 kcal/mol	Joshi et al. (2021)
*Ginkgo biloba* L	Amentoflavone	SARS-CoV-2 M^pro^	Puttaswamy et al. (2020)
*Vitis vinifera*	δ-Viniferin	Interacting ability of δ-viniferin with M^Pro^, RdRp, and hACE-2 suggests its high potential as a multi-target directed ligand against SARS-CoV-2	Joshi et al. (2021)
*Justicia adhatoda*	Anisotine	Anisotine potentially inhibits the spike protein and M^pro^	Kar et al. (2020)
*Swertia chirata*	Amarogentin	Amarogentin potentially inhibits RdRp	
*Psorothamnus arborescens*	3,3-Dimethylallyl isofavone	Binds with 3CL^pro^	Tahir Ul Qamar et al. (2020)
*Myrica cerifera*	Myricitrin	Binds with 3CL^pro^	
*Schisandra sphenanthera*	Excavatolide M cembranolide durumolide K	Potential inhibitor of TMPRSS2	Rahman et al. (2020)
*Aloe barbadensis*	Rhein	Binds with M^pro^	Narkhede et al. (2020)
*Berberis aristata*	Berberine	Binds with M^pro^	
*Moringa oleifera*	Isorhamnetin, kaempferol, apigenin, rutinoside, and vitexin	Inhibits SARS-CoV-2 M^pro^	(Mathpal et al., 2021; Sen et al., 2021)
*Scutellaria baicalensis* Georgi	Baicalin and baicalein	Baicalein showed stronger binding affinity than baicalin to inhibit RdRp	Zandi et al. (2021)
Propolis *Baccharis dracunculifolia*	Limonin, quercetin, and kaempferol	Inhibits RdRp, also inhibit binding with spike protein TMPRSS2 and ACE2	Berretta et al. (2020)
*Uncaria tomentosa* (Cat’s claw)	Proanthocyanidins, epicatechin, proanthocyanidin B2, B4, proanthocyanidin C1, speciophylline, uncarine F, and cadambine	Inhibits M^pro^, 3CL^pro^, interfere ACE2, and spike protein binding	Yepes-Pérez et al. (2020)
*Uncaria tomentosa* (Cat’s claw)	Hydroalcoholic extract of *U. tomentosa* stem bark	Inhibits the release of infectious particles, reducing the cytopathic effect on Vero E6 cells	Yepes-Perez et al. (2021)
*Scutellaria barbata* D. Don	Scutellarin, baicalein luteolin, naringenin, and wogonin	Effectively inhibits M^pro^ and TMPRSS2 activity *in vitro*	Huang et al. (2021)
Citrus fruits	Hesperidin	More interactive with the SARS-CoV-2 PR protein	Kodchakorn et al. (2020)
*Sesamum indicum*	Sesamin		
Peanuts (*Arachis hypogea*)	Resveratrol	Significantly decrease the expression of ACE2, modulating host immune response	(Filardo et al., 2020; de Ligt et al., 2021)

SARS-CoV-2, severe acute respiratory syndrome coronavirus 2; ACE2, angiotensin-converting enzyme-2; 3CLpro, 3 chemotrypsin-like protease; M^pro^, main protease; RdRp, RNA-dependent RNA, polymerase; TMPRSS2, transmembrane protease serine 2.

Because of the antiviral activity of curcumin against various viruses, for example, HIV, Zika virus, herpes simplex virus, Chikungunya virus, hepatitis viruses, and adenovirus (Prasad and Tyagi, 2015; Mounce et al., 2017; Praditya et al., 2019), it may be potentially applied in the management of COVID-19 patients (Ho et al., 2021). A recent molecular docking study revealed that curcumin may inhibit the host entry of SARS-CoV-2 by interfering the viral S protein and host ACE2 receptor protein (Das et al., 2021b). Also, the existing antithrombotic, anti-cytokine, and antifibrotic properties of curcumin may assist in quick recovery of severe COVID-19 patients (Lelli et al., 2017; Wichmann, 2020). Future clinical studies are warranted to develop standard dosages of curcumin to assess possible clinical benefits in patients with COVID-19 (Ho et al., 2021). After compelling previous therapeutic evidence of *N. sativa* and recent molecular docking findings, a recent review suggests that some bioactive compounds of *N. sativa*, for example, *α*-hederin, nigellidine, and thymoquinone, could be used as alternative potential herbal drugs to treat COVID-19 (Islam et al., 2021). Another recent review has updated the current status of the naturally occurring compounds such as alkaloids, terpenes, flavonoids, and benzoquinones from different herbs against SARS-CoV-2 infection and suggested that accurate experimental investigation of these compounds may provide insightful information for the potential therapy of COVID-19 patients (Romeo et al., 2021).

## 13 Genetic Testing for COVID-19 Treatment: Panel of Gene Considerations

Plenty of genes of interest that may either be involved in the severity of COVID-19 progression or may potentially modify the PK/PD profiles of COVID-19 therapeutics, and may therefore potentially affect the safety or effectiveness of these medications have been identified in this review. From reviewing the previous information, we enlisted a panel of genes as two categories: 1) mandate genetic test and 2) recommendations for the genetic test.

### 13.1 Mandate Genetic Test

#### 13.1.1 Mandate Genetic Test for COVID-19 Severity

As described and found evidence in this review, we strongly mandate the genetic test for *HLA*, *ACE2*, and *TMPRSS2* genes for assessing the severity of COVID-19 associated with the genetic variants of these genes.

#### 13.1.2 Mandate Theranostics

Some of the drugs used either as repurposely to combat SARS-CoV-2 infection or used as supportive care for alleviating complications associated with COVID-19 have already well-established evidence for considering pharmacogenomics interventions, and different international pharmacogenomic working groups such as Clinical Pharmacogenetics Implementation Consortium (CPIC) have provided pharmacogenomic-based dosing clinical recommendations as shown in Table 11.

**TABLE 11:** CPIC pharmacogenomic-based dosing guidelines for drug using in COVID-19.

COVID-19 therapeutics (repurposed/supportive care)	Genetic variants	Clinical recommendations	Strength of recommendations	Reference
Atazanavir	UGT1A1*6, *28, *36, *37, *80	NM/IM: Standard therapy	Strong	Gammal et al. (2016)
		PM: Alternative therapy	Strong	
Efavirenz	CYP2B6*4, *6, *18, *22	UM/RM/NM: Standard dose	Strong	Desta et al. (2019)
		IM: Start reduced dose (400 mg/day)	Moderate	
		PM: Start reduced dose (400 mg/day or 200 mg/day)	Moderate	
Clopidogrel	CYP2C19*2, *3, *17	UM/NM: Standard dose	Strong	Scott et al. (2013)
		IM: Alternative antiplatelet, for example, prasugrel or ticagrelor	Moderate	
		PM: Alternative antiplatelet, for example, prasugrel or ticagrelor	Strong	
Warfarin	CYP2C9*2, *3 and *VKORC1* (rs9923231)	Calculate dose for patients carrying these variants based on published validated pharmacogenetic algorithms	Strong	Johnson et al. (2017)
NSAIDs (celecoxib, flurbiprofen, ibuprofen, and lornoxicam)	CYP2C9*2, *3	NM and IM (AS = 1.5): Standard dose	Strong for NM, moderate for IM	Theken et al. (2020)
		IM (AS = 1): Lowest standard dose	Moderate	
		PM: Reduce 25–50% of the lowest recommended dose	Moderate	
Meloxicam	CYP2C9*2, *3	NM/IM (AS = 1.5): Standard dose	Strong for NM, moderate for IM	
		IM (AS = 1): Recommends 50% reduction of the lowest standard dose	Moderate	
		PM: Alternative therapy	Moderate	
Piroxicam and tenoxicam	CYP2C9*2, *3	NM/IM (AS = 1.5): Standard dose	Strong for NM, moderate for IM	
		IM (AS = 1): Alternative therapy	Moderate/optional	
		PM: Alternative therapy is recommended	Moderate/optional	

Here, CPIC, Clinical Pharmacogenetics Implementation Consortium; NM, normal metabolizer; UM, ultrarapid metabolizer; RM, rapid metabolizer; IM, intermediate metabolizer; PM, poor metabolizer; NSAIDs, non-steroidal anti-inflammatory drugs.

At the infancy stage where almost no pharmacogenomics study of COVID-19 therapeutics in this unprecedented health situations, we strongly mandate to undertake at least theranostics for these drug–gene pairs (atazanavir–*UGT1A1*, *ABCB1*, *SLCO1B1*, and *APOA5*; efavirenz–*CYP2B6*; nevirapine–*HLA*, *CYP2B6*, and *ABCB1*; lopinavir–*SLCO1B3* and *ABCC2*; ribavirin–*SLC28A2*; tocilizumab–*FCGR3A*; ivermectin–*ABCB1*; oseltamivir–*CES1* and *ABCB1*; clopidogrel–*CYP2C19* and *ABCB1*, warfarin–*CYP2C9* and *VKORC1*; NSAIDs–*CYP2C9*) in patients with COVID-19 based on the evidence of drug–gene interactions for optimizing the safety or efficacy of COVID-19 therapies.

### 13.2 Recommendations for Theranostics

After evaluating PK properties and low evidence of pharmacogenomic associations, we recommend these drug–gene pairs (remdesivir–*CES1*, *CYP2C8*, *CYP3A4*, and *CYP2D6*; azithromycin–*ABCB1*; losartan–*ABCB1* and *CYP2C9*; lopinavir/ritonavir–*ABCB1*) for further considerations in clinical studies to establish evidence for genetic associations with the safety or effectiveness of COVID-19 medications.

It is reasonable to understand that at the beginning of emergency pandemic situations, clinicians may not be able to prioritize pharmacogenomics intervention issues of COVID-19 drugs; probably that might be one of the best reasons for higher mortality of COVID-19 patients due to not well clinically managed of these patients. Also, although pharmacogenomics is starting to incorporate into routine clinical practice in some parts of the world, for example, United States, Thailand, United Kingdom, and Netherlands, clinicians still are not well-positioned to consider pharmacogenomics recommendations due to either low understanding of this newly evolving area or may not have adequate training regarding the pharmacogenomic uptake in the clinical practice. However, COVID-19 situations are stabilizing slowly, and this is the high time for clinicians/genomics researchers to investigate pharmacogenomics associations of drugs in this clinical condition.

**FIGURE 6:**
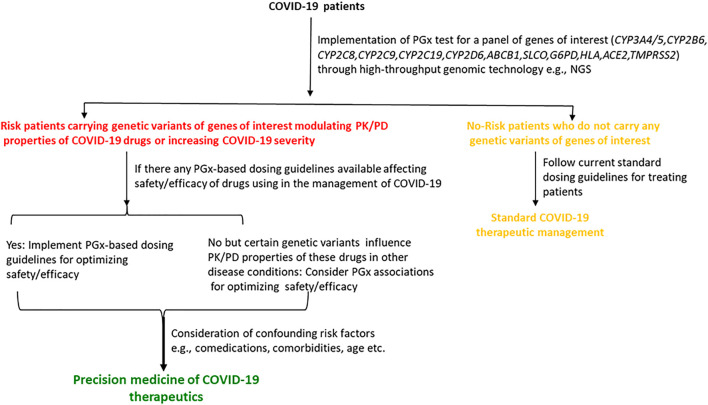
Implementation of precision medicine for drugs used in the management of COVID-19. Here, COVID-19, coronavirus disease-2019; NGS, next-generation sequencing; PK, pharmacokinetics; PD, pharmacodynamics.

Since no pharmacogenomic study assessing the associations of COVID-19 therapeutics with the safety or efficacy has not been either undertaken or published yet, we suggest to consider a panel of genes of interest which have already been discussed above in this review to assess the impacts of pharmacogenomics in COVID-19 therapeutics to establish precision medicine of COVID-19, as illustrated in Figure 6.

## 14 Pharmacogenomics and Precision Medicine for COVID-19 in Thailand

Thai population might be at particular risk for either developing severe COVID-19 due to the *HLA* genetics or developing toxicities/therapeutic ineffectiveness by COVID-19 drugs. This is partly because Thai population has great diversity of *HLA*, transporters, and *CYP* genetic variants. It has been previously reported that ∼25% of the Thai population carried *HLA-DQA1/HLA-DQB1* genetic polymorphisms (Puangpetch et al., 2014; Satapornpong et al., 2020), which might render COVID-19 severity. Minor allele frequencies of the CYP2C9*2 and CYP2C9*3 in Thai population were 0.08 and 5.3%, respectively. Minor allele frequencies of the CYP2C19*2, CYP2C19*3, and CYP2C19*17 in Thai population were 25.6, 2.5, and 1.8%, respectively. Approximately 30% of the *CYP3A4* variant allele was identified in the Thai population as reported elsewhere (Sukprasong et al., 2021). Over 30% Thai population carried *C3435T ABCB1* genetic polymorphisms as revealed in a previous study conducted in Thailand (Sensorn et al., 2013). Overall, it is concluded that considerable proportion of Thai population might be at risk of either severe COVID-19 manifestation or might be at risk of developing toxicities/ineffectiveness of the COVID-19 medications due to carrying these genetic variants. Moreover, the herbs especially andrographolide and the others as described in this review are commonly used in this population; this might also render the risk of toxicities/ineffectiveness of these herbs due to either DHIs or herbogenomics.

## 15 Clinical Perspective

**FIGURE 7:**
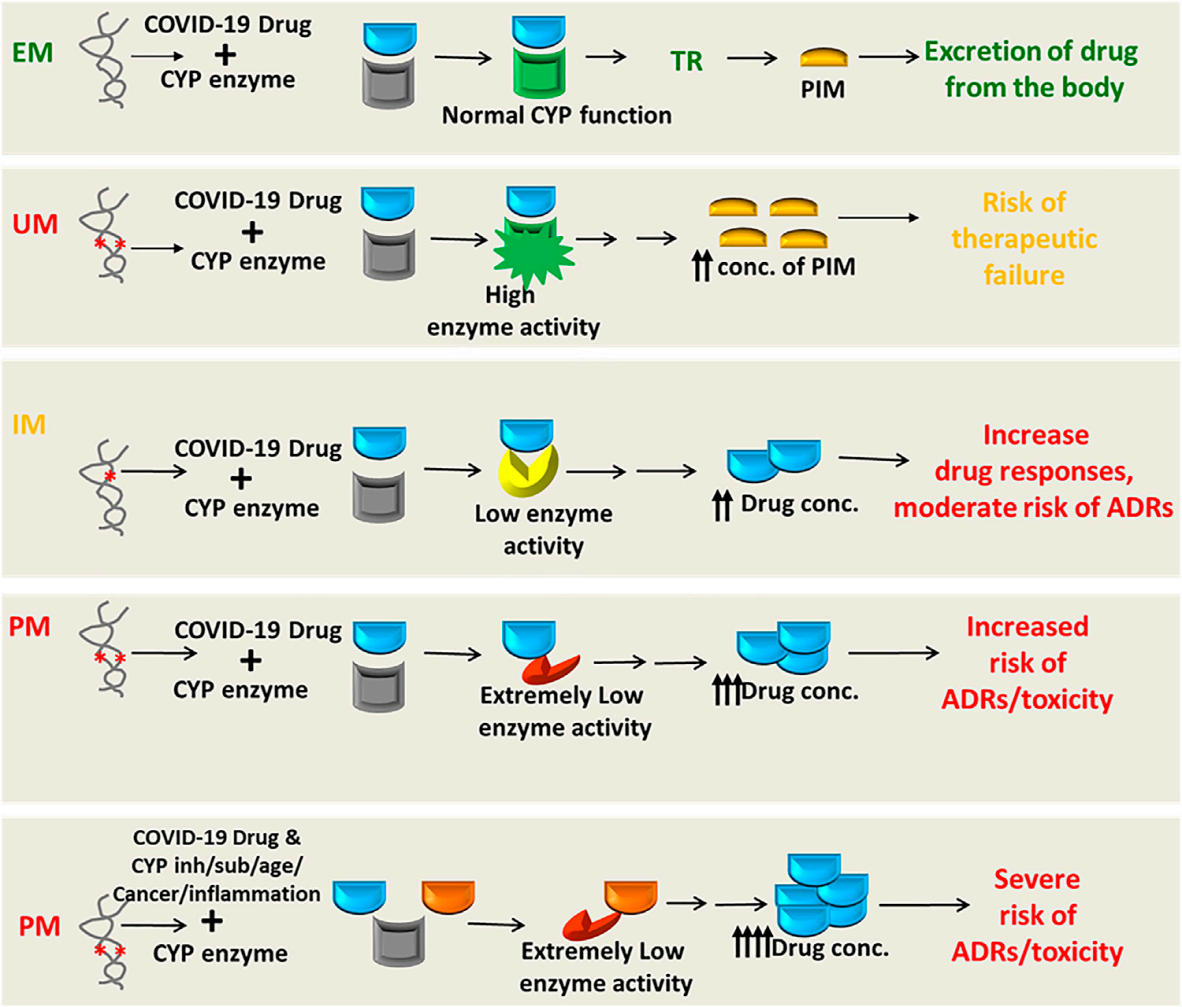
Predictive model of multifactorial DGIs for a COVID-19 drug showing possible effects of *CYP* gene variants representing likely phenotypes associated with the risk of therapeutic failure or ADRs/toxicity. Here, DGIs, drug–gene interactions; COVID-19, coronavirus disease-2019; EM, extensive metabolizer; UM, ultrarapid metabolizer; IM, intermediate metabolizer; PM, poor metabolizer; CYP, cytochrome P450 enzyme; *indicates *CYP* gene variant; Inh, inhibitor; Subs, substrate; TR, therapeutic response; PIM, pharmacologically inactive metabolite; ADRs, adverse drug reactions; conc., concentration.

To our best knowledge, no clinical studies were identified in the literature to date that had assessed either metabolic or transporter genetic variants with the safety or effectiveness of current COVID-19 therapeutics. This is creating evidence impasse and delaying the target for finding appropriate therapeutics to combat COVID-19 successfully. From considering the PK/PD profiles of the current COVID-19 therapeutics under investigation as discussed in this review, it is emerging the needs for assessing genetic associations of the relevant metabolic or transporter genes of interest for optimizing the safety or effectiveness of COVID-19 therapeutics. Future clinical studies or trials are warranted to investigate such genetic associations for the achievement of precision medicine for COVID-19. Since it is well evidenced that the mortality is significantly higher in older people and having comorbidities (Biswas et al., 2021a), DDIs should also be considered in these assessments because of vulnerability to polypharmacy. Ideally, considerations of multifactorial drug–gene interactions (DGIs) of COVID-19 therapeutics may accelerate the development of precision medicine of COVID-19 in the real clinical settings as shown in Figure 7, as established in other therapeutic areas such as antiplatelets (Biswas et al., 2021b).

It is very important to note here that this predictive model has considered only a pharmacologically active drug involving CYP metabolism; however, in case of a prodrug, the effects will be *vice versa*, and this model is also applicable to other genes, for example, transporter genes affecting the safety or efficacy of COVID-19 drugs.

## 16 Conclusion

The global outbreak of SARS-CoV-2 has evolved into an emergent COVID-19 pandemic causing huge morbidity and mortality in the world. Many drugs without establishing clinical effectiveness or tailoring safety are being administered to tackle COVID-19 pandemic situations. A repurposing strategy might be more effective and successful if pharmacogenetic interventions of these drugs are being considered in future clinical studies/trials. Safety and effectiveness of several repurposed drugs currently being used for the management of COVID-19 may be affected by the *CYP*/transporter genetic variants. From reviewing the current evidence of pharmacogenetic of these drugs in either COVID-19 or other diseases, we strongly mandate to undertake pharmacogenetic assessment for at least these drug–gene pairs (atazanavir–*UGT1A1*, *ABCB1*, *SLCO1B1*, and *APOA5*; efavirenz–*CYP2B6*; nevirapine–*HLA*, *CYP2B6*, and *ABCB1*; lopinavir–*SLCO1B3* and *ABCC2*; ribavirin–*SLC28A2*; tocilizumab–*FCGR3A*; ivermectin–*ABCB1*; oseltamivir–*CES1* and *ABCB1*; clopidogrel–*CYP2C19* and *ABCB1*, warfarin–*CYP2C9* and *VKORC1*; NSAIDs–*CYP2C9*) in patients with COVID-19 for advancing precision COVID-19 therapeutics by optimizing the safety or effectiveness of these drugs.

Although it is very unlikely that there are almost no pharmacogenetic data for COVID-19 drugs, from inferring the PK/PD properties and some pharmacogenetic evidence of these drugs in other diseases/clinical conditions, it is highly likely that pharmacogenetic associations are also feasible in at least some COVID-19 drugs currently being administered as shown in this review and should be considered in future clinical studies/trials. Molecular docking and computational studies are promising to achieve new COVID-19 therapies as shown in this review. Current situation in the discovery of anti-SARS-CoV-2 agents at four important targets from *in silico* studies has been described and summarized in this review. Although naturally occurring compounds from different herbs against SARS-CoV-2 infection are favorable, accurate experimental investigation of these compounds is warranted to provide insightsful information. Moreover, clinical considerations of DDIs and DHIs of the existing repurposed drugs along with pharmacogenetic (e.g., efavirenz and *CYP2B6*) and herbogenetic (e.g., andrographolide and *CYP2C9*) interventions, collectively called multifactorial drug-gene interactions (DGIs), may further accelerate the development of precision COVID-19 therapies in the real-world clinical settings.
